# Multicomponent Online Intervention Improves Sarcopenia-Related Traits Following Long-Term Metabolic Bariatric Surgery: A Randomized Clinical Trial

**DOI:** 10.1007/s11695-026-08742-x

**Published:** 2026-05-22

**Authors:** Gabriela Sousa de Oliveira, Ariene Silva do Carmo, Simone Gonzaga do Carmo, Patrícia Borges Botelho, Ricardo Moreno Lima, Kenia Mara Baiocchi de Carvalho

**Affiliations:** 1 Graduate Program in Human Nutrition, University of Brasília, Brasilia, Brazil; 2 State University of Campinas, Campinas, Brazil

**Keywords:** Metabolic bariatric surgery, Telemedicine, Multicomponent intervention, Sarcopenic obesity

## Abstract

**Introduction:**

Telemedicine provides outcomes comparable to in-person follow-up after Metabolic Bariatric Surgery (MBS), but evidence on multicomponent online interventions in the late postoperative period remains scarce. This study evaluated the effects of a supervised online multicomponent program on sarcopenia risk and related components in adults 2–7 years after MBS.

**Methods:**

In this randomized controlled trial, participants were assigned to either the multicomponent online group (*n* = 79) or control group (*n* = 60). The intervention combined nutritional counseling, delivered through synchronous group sessions and pre-recorded videos addressing behavioral strategies; and supervised exercise training including aerobic and resistance components. Assessments included body composition, phase angle, handgrip strength, sit-to-stand test, functional performance, resting energy expenditure, and biochemical analyses. Sarcopenia risk was assessed based on phase angle. Analyses used generalized estimating equations (intention-to-treat, *p* < 0.05).

**Results:**

After 12 weeks, the multicomponent online group (mean age 40.1 ± 7.5 years; BMI 29.6 ± 5.1 kg/m²; 3.9 ± 1.7 years post-surgery), compared with controls (mean age 40.6 ± 8.1 years; BMI 29.2 ± 5.0 kg/m²; 3.9 ± 1.6 years post-surgery) showed improvements in phase angle (β = 0.15; 95% CI: 0.02; 0.29; *p* = 0.025), handgrip strength/body weight (β = 0.03; 95% CI: 0.001; 0.06; *p* = 0.043), sit-to-stand time test (β = − 1.22; 95% CI: − 2.30; − 0.14; *p* = 0.027). Regarding biochemical outcomes, C-reactive protein and HOMA-β levels decreased, while serum creatinine and glucose increased within clinical reference ranges (*p* < 0.05). Compared to controls, the intervention group had a significantly lower likelihood of sarcopenia risk after 12 weeks (OR = 0.41; 95% CI: 0.19; 0.87; *p* = 0.020).

**Conclusion:**

A supervised online multicomponent intervention improved muscle strength and lowered sarcopenia risk after MBS, highlighting the potential of remote multicomponent strategies as a feasible approach to preserve muscle health and prevent sarcopenia in long-term bariatric care.

**Supplementary Information:**

The online version contains supplementary material available at 10.1007/s11695-026-08742-x.

## Key Points

Multicomponent online intervention enhanced muscle strength in the late post-MBS.

Multicomponent online intervention showed reduced sarcopenia risk.

Multicomponent online protocols are feasible for long-term MBS follow-up.

**Supplementary Information:**

The online version contains supplementary material available at 10.1007/s11695-026-08742-x.

## Introduction

 Metabolic and bariatric surgery (MBS) is the most effective treatment for severe obesity, producing substantial weight loss, reductions in all-cause mortality, remission of associated medical problems, and improvements in functional capacity and quality of life [1–4]. Nevertheless, some degree of recurrent weight gain frequently emerges after the second postoperative year [5], driven by metabolic adaptations [6], inadequate diet, physical inactivity, and lack of follow-up care [7–9]. These factors may adversely affect body composition, as postoperative weight gain is often marked by disproportionate fat mass accretion relative to lean mass, favoring the development of sarcopenic obesity and impairing physical function [10]. This condition not only deteriorates functional outcomes but also decreases energy expenditure related to fat-free mass, potentially compromising long-term weight control [11].

Given the multifactorial nature of obesity [12], effective management depends on comprehensive behavioral change strategies [13]. Multidisciplinary interventions delivered through in-person formats, integrating nutrition, physical activity counseling, and mindfulness-based strategies, yield superior benefits for anthropometric outcomes, eating behavior, quality of life, and psychological health compared with isolated approaches in adults with obesity [14, 15].

The COVID-19 pandemic accelerated the adoption of telemedicine, expanding access to remote healthcare [16], reducing costs [17] and increasing patient satisfaction [18]. Remote isolated interventions guided by healthcare professionals have shown results comparable to in-person care in the follow-up of patients after MBS [19]. However, very few studies have investigated online multicomponent interventions in the late postoperative period, defined in this study as 2–7 years after surgery. Research has mainly focused on the early months after surgery, when weight loss occurs largely independent of professional guidance. Therefore, further research is required to address the late postoperative period (≥ 2 years after MBS), when recurrent weight gain and sarcopenia become more prevalent [19]. Taken together, these findings suggest that combining the accessibility of telemedicine with the established benefits of multidisciplinary, multicomponent care may represent a logical and promising strategy for long-term postoperative management after MBS.

To our knowledge, few studies have evaluated multicomponent online interventions in the late postoperative period after MBS, and none have specifically examined the combined effects of supervised online exercise with nutritional counseling and behavioral strategies on sarcopenia-related outcomes, all monitored by healthcare professionals. Given the need for effective strategies to improve muscle-related outcomes in patients at risk of recurrent weight gain after MBS, this study aimed to assess the effects of a multicomponent online intervention program on sarcopenia risk and related health components in the late postoperative period after MBS. We hypothesized that the intervention would reduce sarcopenia risk and lead to improvements in metabolic outcomes compared with the control condition.

## Methods

### Protocol

This randomized controlled trial was prospectively registered in the Brazilian Registry of Clinical Trials under the identifier RBR-4pdv53d (https://ensaiosclinicos.gov.br/rg/RBR-4pdv53d; August 12, 2023) and conducted in the laboratories of the University of Brasília. The study design followed the guidelines of the Consolidated Standards of Reporting Trials [20].

### Public Involvement and Eligibility Criteria

To recruit adults of both sexes, 2–7 years post-Roux-en-Y gastric bypass (RYGB) or sleeve gastrectomy, we used posters in clinics/hospitals and on social media. The postoperative time determined for this study represents a late postoperative phase, marked by discontinued clinical follow-up.

Exclusion criteria included regular exercise or external nutrition counseling within the preceding three months, decompensated chronic diseases, conditions limiting exercise, pregnancy or breastfeeding, psychiatric disorders or psychotropic drug use (e.g., diazepam, lorazepam, midazolam, phenytoin, carbamazepine, amitriptyline), use of weight-loss medications (e.g., liraglutide, semaglutide, sibutramine, orlistat, mazindol, anfepramone), use of weight-loss medications, amputations, and lack of access to a mobile device or computer.

Sample size was estimated with G*Power^®^ 3.1 for repeated measures (time × group interaction), assuming a medium effect size (f = 0.25) according to Cohen [21], a 5% significance level, and 80% power, resulting in a minimum of 100 participants. The sample size was calculated assuming a conventional moderate effect size, given the exploratory nature of the study and the presence of multiple outcomes. Accounting for an anticipated 25% dropout rate, the final target sample size was set at 125 individuals [22].

### Trial Design and Setting

This study was a two-arm, parallel-group randomized controlled trial with a 1:1 allocation ratio. Recruitment was conducted in three sequential waves due to logistical constraints, specifically to ensure high-quality supervision and adequate trainer-to-participant ratios during the online exercise sessions. For each wave, a simple randomization sequence was independently generated using Research Randomizer^®^ (https://www.randomizer.org/).

Although formal allocation concealment was not implemented, a strict chronological procedure was adopted to minimize selection bias. The randomization sequence was neither pre-generated nor accessible in advance. Instead, the researcher accessed the tool only after each participant had been formally enrolled and baseline assessments had been completed. This “just-in-time” disclosure protocol ensured that group assignments remained unpredictable during the recruitment phase, even though the same investigator was responsible for both generating the allocation sequence and study management.

The intervention group received 12 weeks of online nutritional counseling and supervised exercise. Controls were placed on a waiting list to receive the same program after completing the study. To minimize confounding variables, participants were instructed to maintain their baseline dietary habits and medication regimens throughout the trial. Any alterations in these factors, or the initiation of outside physical activities, were to be reported immediately to the research team. Adherence to these guidelines was monitored via standardized questionnaires and during the weekly supervised sessions.

Assessments were conducted in person at baseline (randomization) and after 12 weeks. Intervention sessions were delivered remotely via videoconference and supervised by qualified physical education professionals and nutritionists, with an average of eight participants per session. All participants underwent a medical evaluation prior to the start of the study to confirm eligibility and ensure safety during physical exercise.

Adherence was monitored by session attendance. It was observed that, among the participants who withdrew from the study, all had attendance in either the nutritional counseling or exercise sessions rates below 70% (Suppl Table 1).

### Nutritional Counseling with a Behavioral Approach

Participants randomized to the Multicomponent online group participated in a group-based nutritional counseling program with a behavioral approach, based on the Brazilian Cardioprotective Diet [23], developed by Brazil’s Ministry of Health. It is a structured nutritional resource aimed at individuals with diet-related cardiovascular risk factors, such as obesity [23]. Participants attended 60-minute synchronous online group sessions once a week, supported by 13 pre-recorded videos with practical activities (~ 20 min), delivered via a mobile messaging app. In the synchronous sessions, they shared their personal experiences and discussed the practical tasks assigned during the study. Details on content and activities are provided in the supplementary material (Suppl Tables 2 and 3).

### Physical Exercise Program

The online exercise program was performed three times per week on non-consecutive days, with ~ 60-minute sessions supervised in real time by qualified physical education professionals. The protocol was designed and adapted in accordance with the American College of Sports Medicine (ACSM) guidelines for individuals with obesity [24], with a focus on cardiorespiratory fitness and muscular strength through home-based activities. Each session included warm-up; aerobic exercise; resistance training using bodyweight, dumbbells, and chairs; and a cool-down period. Participants received equipment (dumbbells of varying loads and exercise mats) prior to initiation. Training load was monitored using the OMNI-Resistance Exercise Scale (OMNI-RES) [25] and progressively increased throughout the intervention. Participants were instructed to maintain their usual routines and avoid other structured exercise programs. The complete exercise protocol is detailed in the supplementary material (Suppl Table 5 for supervised remote physical exercise program and Table 5 for description of the circuits, in addition to a schematic illustration of the aerobic and resistance exercises proposed in the study training protocol).

Adverse events and unintended effects were systematically monitored throughout the intervention period through participant self-report and supervision by trained professionals during the online sessions.

### Sociodemographic and Surgical Data

Sociodemographic characteristics were collected using a questionnaire comprising both closed- and open-ended questions regarding sex, date of birth, education level, type of MBS, and date of the surgery procedure.

### Anthropometric, Body Composition Assessment, and Clinical Data

Anthropometric and body composition assessments were performed with participants barefoot and in light clothing. Height was measured with a wall-mounted stadiometer (AlturaExata^®^, Brazil), and body weight and composition with a multifrequency bioelectrical impedance analyzer (InBody720^®^, Biospace, Korea). To standardize conditions on the day of clinical assessments, participants fasted for at least 8 h, avoided caffeine and physical activity for 24 h, emptied the bladder, removed metal accessories, and, when applicable, were assessed outside the menstrual period. Measured parameters included body weight (kg), fat-free mass (FFM, kg), skeletal muscle mass (SMM, kg), fat mass (kg), body fat percentage, and phase angle (PhA, °). PhA (°) was derived from reactance (Xc) and resistance (R) at 50 kHz using the formula: arctangent (Xc/R) × (180/π). Body Mass Index (BMI) (kg/m²) was calculated based on weight and height. Excess weight loss (EWL) > 50% and total weight loss (TWL) > 20% were considered satisfactory [26], whereas recurrent weight gain was defined as an increase > 10% from the lowest postoperative weight [27]. Ideal weight corresponded to a BMI of 25 kg/m².

### Assessment of Sarcopenia Risk

Sarcopenia risk was assessed based on the previously calculated phase angle (PhA). The cut-off point applied for sarcopenia risk screening (PhA < 5.25^o^) corresponds to the upper limit of the proposed threshold reported in a recent systematic review with meta-analysis, which proposed PhA as a robust screening tool for sarcopenia [28]. In this study, we used phase angle as a screening tool for sarcopenia risk due to its strong correlation with muscle mass and function [28], acknowledging that it does not constitute a formal diagnosis of sarcopenia according to international criteria.

### Assessment of Obesity Classification Criteria

Obesity classification was determined based on body fat percentage obtained through Bioelectrical Impedance Analysis (BIA), according to the cut-off values recommended by the European Society for Clinical Nutrition and Metabolism (ESPEN) and the European Association for the Study of Obesity (EASO) [29]. According to the reference values for the white population established by Gallagher et al. [30], obesity was defined as a body fat percentage > 25%, > 28%, and > 30% for men and > 35%, > 38%, and > 42% for women in the age groups of 20–39, 40–59, and 60–79 years, respectively. These reference values account for sex, age, BMI, and ethnicity to appropriately evaluate fat mass [30].

### Muscle Strength Measurement

Upper limb muscle strength was assessed using the handgrip strength (HGS) test, performed with a JAMAR^®^ isometric dynamometer (Lafayette Instrument Company, Lafayette, IN, USA) applied to the dominant arm. Three consecutive measurements were taken at one-minute intervals, and their average was used, as previously described [31].

Lower limb strength was evaluated using the five repetitions Sit-to-Stand Test (STS_5r). Upon verbal command, participants were asked to stand up fully and sit back down on a standard chair five times as quickly as possible without using their hands for support. The total time taken to complete the five repetitions was recorded and used as the final measure of lower limb strength [32]. The variables thus obtained were STS_5r (s) and dominant HGS relative to body weight (kgf/kg).

### Physical Performance Parameters

Physical performance parameters were assessed using the Timed Up and Go (TUG) test and 10-Meter Walk Test (10MWT) [33, 34]. For the TUG, after instructions and a familiarization trial, participants stood up from a standard chair, walked 3 m at their fastest safe pace, turned, returned, and sat down. Three attempts were performed with 60 s rest, and the shortest time was recorded. For the 10MWT, participants walked a 10 m path at usual pace; the first and last 2 m were used for acceleration and deceleration, and timing was applied to the central 8 m. Two trials were conducted, and the best time was considered. All assessments were performed by the same trained evaluator.

### Resting Energy Expenditure Assessment

Resting energy expenditure (REE) was assessed by open-circuit indirect calorimetry (Vmax 29^®^, SensorMedics, USA) [35, 36]. After a 10-min rest in a quiet, dimly lit room, participants remained awake and breathed normally through a ventilated canopy for 30 min. Gas analyzers were calibrated before each test. REE was calculated based on the final 20 min using the Weir equation [37], considering steady state as VO₂ and VCO₂ variations < 10% [38], and expressed as absolute REE (kcal/day), REE per body weight (kcal/kg), REE per FFM (kcal/kg), and respiratory quotient.

### Laboratory Blood Analysis

Fasting blood samples (8–14 h) were collected in the same week as the other assessments and analyzed using standardized laboratory methods. Biomarkers included glucose (hexokinase), insulin (chemiluminescence), glycated hemoglobin (immunoturbidimetric), total cholesterol (esterase/oxidase), HDL-cholesterol (enzymatic colorimetric), LDL-cholesterol (elimination/catalase), triglycerides (oxidase/peroxidase), C-reactive protein (latex-enhanced immunoturbidimetric method), and serum creatinine (kinetic colorimetric). Insulin resistance (HOMA-IR) and beta-cell function (HOMA-β) indices were calculated as previously described [39].

For 24-hour urinary creatinine excretion (CER), participants collected all urine for 24 h after discarding the first morning void. Completeness was verified by creatinine excretion relative to body weight (cut-offs: ≥20 mg/kg for men and ≥ 15 mg/kg for women < 50 y; ≥10 mg/kg and ≥ 7.5 mg/kg for men and women ≥ 50 y, respectively). CER (mmol/day) was calculated by multiplying urinary creatinine concentration by total volume. The creatinine height index (%CHI) was derived from total urinary creatinine relative to expected values by sex and height; %CHI > 75% indicated absence of muscle mass depletion.

### Physical Activity Assessment

Physical activity was assessed as a control variable using the 24-hour Physical Activity Recall (R24hPA)—an instrument adapted and validated for the Brazilian population [40]. The recall was administered exclusively at baseline (one week prior to the intervention) and at the final follow-up (one week after the intervention period ended). To ensure that the measurements reflected habitual activity without interference from study protocols, the R24hPA was administered on two non-consecutive days that did not coincide with the clinical assessment dates (which required a 24-hour exercise restriction). Consequently, the structured online exercise sessions were not included in the recalls, as these assessments took place outside the intervention timeframe. Participants reported all activities lasting ≥ 10 min in hourly intervals, including duration and intensity. Activities were converted to metabolic equivalent (MET) values using the Compendium of Physical Activities [41], and daily energy expenditure was expressed as the Physical Activity Level (PAL). Participants were classified as inactive (PAL < 1.7) or active (PAL ≥ 1.7) [42].

### Dietary Intake Assessment

Usual energy and protein intake were assessed as control variables at baseline and after 12 weeks, using two non-consecutive 24-hour dietary recalls at each time point, following the 5-step Multiple Pass Method [43]. A photographic food atlas with images of common Brazilian foods, utensils, and household measures was used to aid portion size estimation [44]. Reported intakes were converted into grams or milliliters using BRASIL NUTRI^®^ software, and usual intake adjusted for intra-individual variability was estimated with the Multiple Source Method (MSM^®^) [45].

The primary comparison of this study was the presence versus absence of the online multicomponent intervention. Other than the intervention factor, both groups underwent the same baseline evaluation, were assessed over the same 12-week period, and did not participate in other structured programs, ensuring that observed differences could be attributed to the intervention.

### Statistical Analysis

Primary outcomes were sarcopenia risk and body composition; secondary outcomes included obesity classification, limb muscle strength, physical performance, biochemical parameters, and resting energy expenditure. Analyses following a modified intention-to-treat approach were used, including all randomized participants with at least one post-randomization measurement available, examining group-by-time interactions. An intention-to-treat analysis was employed to preserve the benefits of randomization, minimize biases arising from losses and protocol deviations, and provide a more conservative estimate that better reflects the effectiveness of the intervention under real-world conditions. Additionally, factors associated with intervention attendance were explored.

Descriptive analysis included the calculation of absolute and relative frequencies for categorical variables, and mean, standard deviation, and 95% confidence intervals (CI) for quantitative variables. For group comparisons at baseline, Student’s t-test, Mann–Whitney U test, and Chi-square or Fisher’s exact tests were applied, where appropriate. Correlations between continuous variables were assessed via Pearson or Spearman tests.

Intervention effects were analyzed using generalized estimating equations (GEE). For quantitative dependent variables, the working correlation matrix used was an unstructured and robust estimator covariance matrix. The variables were treated as normal distribution, with a connection identity function. Unstandardized Coefficients and their respective confidence intervals (95% CI) were calculated. For categorical dependent variables, the working correlation matrix used was exchangeable and robust estimator covariance matrix. The variables were treated as binomial distribution, with a connection logit function. Odds ratio and their respective confidence intervals (95% CI) were calculated. GEE model structure was adjusted for baseline values. For multiple comparisons, the Bonferroni post-hoc test was used. Significance was set at *p* < 0.05. Analyses were conducted using Statistical Package for the Social Sciences 25.0 (IBM Corp., Armonk, NY, USA).

## Results

**Fig. 1:**
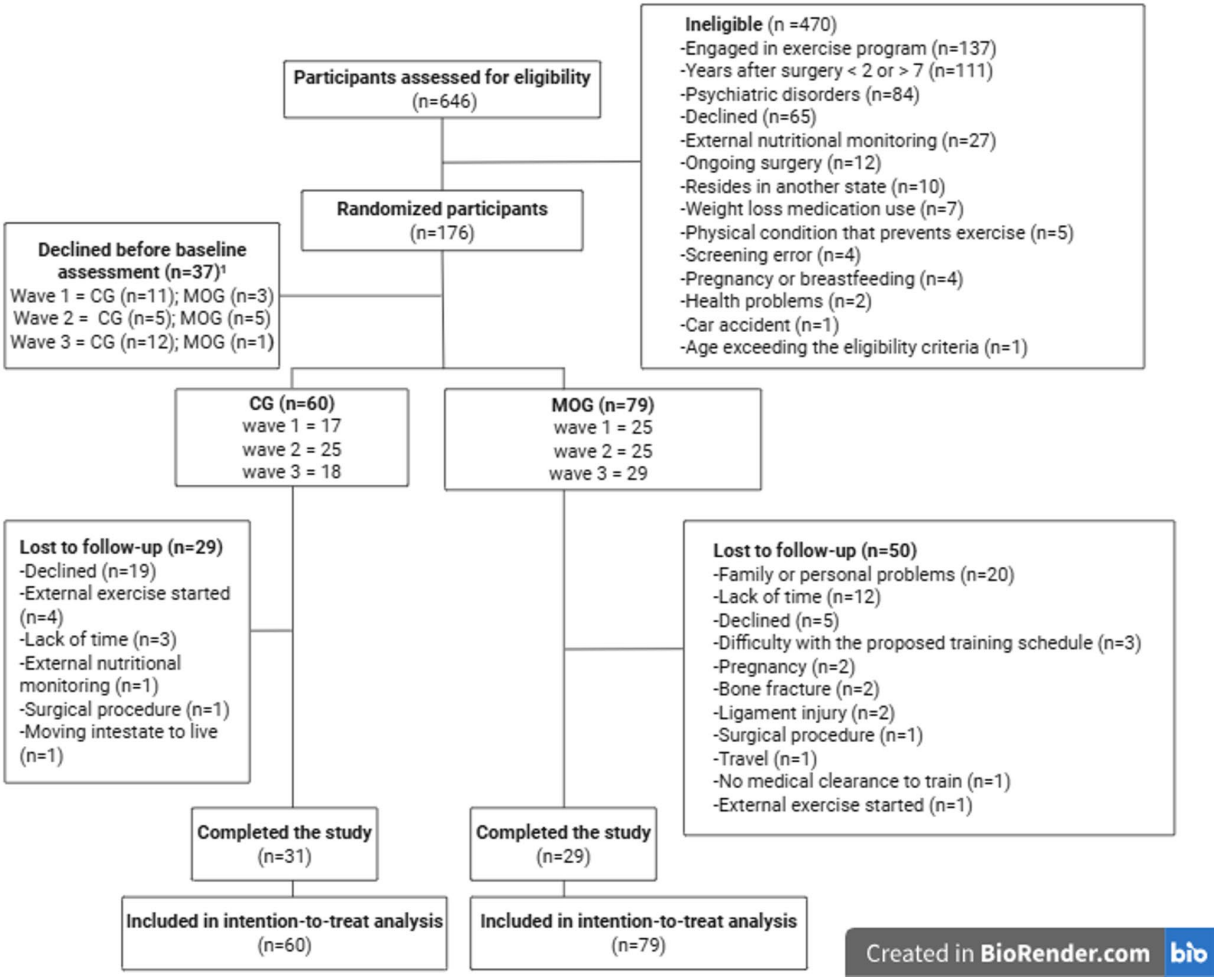
Flowchart of participant allocation process, sample randomization, and dropout rates at each study stage. ^1^Randomized participants who did not complete baseline assessment and therefore had no data available for inclusion in the analyses. CG control group; MOG Multicomponent online group

Randomization was conducted in three separate waves over a 20-month data collection period (September 2023 to January 2025), using a 1:1 allocation ratio between the multicomponent online and control groups. The number of participants randomized in each of the three waves is illustrated in Fig. 1. Of the 139 individuals enrolled in the study, 60 completed the full study protocol. No significant differences were observed in demographic or clinical characteristics between participants who completed the study (*n* = 60) and those who withdrew (*n* = 79) (supplementary material – Table 1).

Baseline characteristics were similar between groups. Most participants were women (~ 90%) who had undergone RYGB (> 90%), with a mean age of 40 years and a mean BMI of approximately 30 kg/m² in both groups. Although participants achieved substantial excess weight loss and total weight loss, more than half experienced weight regain (61%). The mean MET-h/day value indicated an inactive physical activity level, and the average usual energy and protein intake (~ 23 kcal/kg/day and ~ 1.1 protein g/kg/day) were similar between groups (Table 1). There were no significant changes in energy or protein intake during the intervention period (data not shown).

**Table 1:** Baseline demographic and clinical characteristics of participants in the late postoperative period of metabolic bariatric surgery, according to study groups (Control vs. Multicomponent online group)

Variables	All sample (*n* = 139)	Multicomponent online group (*n* = 79)	Control group (*n* = 60)	*p* value
Female [n (%)]	126 (90.6)	70 (88.6)	56 (93.3)	0.343^1^
Age (years)	40.2 ± 7.7	40.1 ± 7.5	40.6 ± 8.1	0.956^2^
Education level (years of study)	14.5 ± 2.8	14.3 ± 2.9	14.8 ± 2.6	0.602^2^
RYGB surgical procedure [n (%)]	131 (94.2)	74 (93.7)	57 (95.0)	0.520^3^
Years after surgery (years)	3.9 ± 1.6	3.9 ± 1.7	3.9 ± 1.6	0.882^2^
Preoperative body mass index (kg/m^2^)	42.1 ± 5.6	41.8 ± 5.4	42.5 ± 6.0	0.290^2^
Current body mass index (kg/m^2^)	29.4 ± 5.0	29.6 ± 5.1	29.2 ± 5.0	0.711^2^
Excess weight loss (%)	78.1 ± 25.4	76.8 ± 25.6	79.9 ± 25.1	0.469^4^
Total weight loss (%)	38.6 ± 7.1	37.9 ± 6.1	39.4 ± 8.2	0.211^4^
Recurrent weight gain^5^ [n (%)]	85 (61.1)	50 (63.3)	35 (58.3)	0.503^1^
Mean of recurrent weight gain (*n* = 85) (%)	14.0 ± 9.4	14.1 ± 9.3	13.9 ± 9.6	0.902^2^
Mean of usual energy intake (kcal/day)	1800 ± 484	1856 ± 487	1730 ± 479	0.116^2^
Mean of usual protein intake (g/day)	85.5 ± 22.4	87.5 ± 24.8	82.5 ± 19.4	0.216^2^
Mean of MET-h/day	1.37 ± 0.17	1.36 ± 0.16	1.39 ± 0.18	0.205^2^

^1^ Chi-square test; ^2^ Mann–Whitney U test; ^3^ Fisher´s exact test ^4^ Student’s t test for independent samples; ^5^ Recurrent weight gain when > 10% of the lowest weight obtained in the postoperative period. MET-hour/day metabolic equivalent of task-hour/day; RYGB Roux-en-Y gastric bypass

Within the multicomponent online group, mean attendance was 57.5% for the online exercise training, 76.8% for the nutritional counseling, and 62.6% for both interventions. A positive correlation was observed between years of education level and total study attendance (%) (*r* = 0.231, *p* = 0.047), as well as between age and total attendance (%) (*r* = 0.379, *p* = 0.001) (data not shown).

Laboratory parameters were within normal ranges at baseline and post-intervention, with no differences observed between groups at baseline. Post-intervention between-group comparisons revealed significantly higher mean glucose levels (β = 3.41; 95% CI: 0.29; 6.53, *p* = 0.032), lower mean C-reactive protein levels (β = −5.09; 95% CI: −9.30; −0.89, *p* = 0.018), elevated mean creatinine levels (β = 0.03; 95% CI: 0.001; 0.06, *p* = 0.049), and reduced mean HOMA-β values (β = −60.46; 95% CI: −112.4; −8.48, *p* = 0.023) in the multicomponent online group compared with the control group (Table 2).

**Table 2:** Effect of multicomponent online intervention on laboratory parameters in individuals in the late postoperative period of metabolic bariatric surgery

Variables	Multicomponent online group		Control group		β^1^ (95% CI)	*p* value^2^
	Baseline	After 12 weeks	Baseline	After 12 weeks		
	Mean (95% CI)	Mean (95% CI)	Mean (95% CI)	Mean (95% CI)		
Glucose (mg/dL)	80.2 (78.6; 81.7) ^aA^	84.2 (82.5; 86.0) ^bB^	79.6 (78.1; 81.0) ^aA^	80.3 (77.4; 83.1) ^aA^	3.41 (0.29; 6.53)	**0.032**
Basal insulin (µUI/mL)	6.4 (5.6; 7.1) ^aA^	6.3 (5.5; 7.1) ^aA^	6.5 (5.3; 7.8) ^aA^	8.1 (4.5; 11.8) ^aA^	−1.66 (−5.18; 1.85)	0.354
HOMA-IR	1.3 (1.1; 1.4) ^aA^	1.3 (1.1; 1.5) ^aA^	1.3 (1.0; 1.6) ^aA^	1.8 (1.0; 2.5) ^aA^	−0.44 (−1.17; 0.29)	0.236
HOMA-β	141.4 (123.7; 159.0) ^aA^	111.4 (92.3; 130.4) ^aB^	142.8 (116.1; 169.5) ^aA^	173.3 (112.7; 233.8) ^aA^	−60.46 (−112.4; −8.48)	**0.023**
HbA1c (%)	5.37 (5.28; 5.45) ^aA^	5.41 (5.33; 5.49) ^aA^	5.38 (5.30; 5.45) ^aA^	5.44 (5.35; 5.52) ^aB^	−0.02 (−0.10; 0.07)	0.701
Total cholesterol (mg/dL)	161.8 (155.0; 168.6) ^aA^	160.2 (154.0; 166.4) ^aA^	158.7 (153.4; 164.1) ^aA^	164.2 (156.0; 172.4) ^aA^	−7.04 (−15.66; 1.58)	0.110
HDL-c (mg/dL)	58.1 (54.8; 61.3) ^aA^	56.7 (53.3; 60.2) ^aA^	56.5 (53.0; 60.0) ^aA^	57.0 (53.2; 60.9) ^aA^	−1.89 (−4.84; 1.07)	0.211
LDL-c (mg/dL)	90.1 (84.3; 95.8) ^aA^	89.0 (83.0; 95.0) ^aA^	88.5 (82.4; 94.5) ^aA^	93.3 (85.5; 101.1) ^aA^	−5.92 (−13.35; 1.51)	0.118
Triglycerides (mg/dL)	79.5 (69.0; 90.0) ^aA^	79.9 (70.2; 89.5) ^aA^	69.9 (64.1; 75.6) ^aA^	70.7 (63.7; 77.8) ^aA^	−0.47 (−13.57; 12.63)	0.944
Serum creatinine (mg/dL)	0.67 (0.64; 0.70) ^aA^	0.71 (0.67; 0.74) ^aB^	0.66 (0.63; 0.69) ^aA^	0.67 (0.62; 0.71) ^aA^	0.03 (0.001; 0.06)	**0.049**
C-reactive protein (mg/dL)	3.09 (0.35; 5.82) ^aA^	0.60 (0.34; 0.85) ^aA^	0.51 (0.26; 0.77) ^aA^	3.12 (−0.07; 6.31) ^aA^	−5.09 (−9.30; −0.89)	**0.018**
CER (mmol/24-h)	13.9 (12.5; 15.4) ^aA^	18.8 (12.3; 25.3) ^aA^	12.0 (11.1; 13.0) ^aA^	15.5 (9.5; 21.6) ^aA^	1.34 (−8.10; 10.79)	0.780
CHI (%)	81.9 (70.3; 93.6) ^aA^	99.3 (78.6; 120.0) ^aA^	88.8 (71.9; 105.6) ^aA^	92.3 (70.6; 114.1) ^aA^	13.80 (−29.52; 57.12)	0.532

^1^ Unstandardized beta between group comparison at the post-intervention time. ^2^ Generalized estimating equations adjusted for the baseline. CER creatinine excretion rate; CI confidence interval; CHI creatinine high index; HDL-c high density lipoprotein; HbA1c glycated hemoglobin; LDL-c low density lipoprotein

Lowercase letters indicate between-group comparisons at each time point. Uppercase letters indicate within-group comparisons over time

No baseline differences were observed between the control and multicomponent online groups in anthropometric measures, body composition, muscle strength, physical performance, or resting energy expenditure. Post-intervention between-group comparisons revealed significantly higher mean PhA (β = 0.15; 95% CI: 0.02; 0.29, *p* = 0.025), higher mean handgrip strength/body weight (β = 0.03; 95% CI: 0.001; 0.06, *p* = 0.043), and shorter mean time to complete the STS_5r test (β = −1.22; 95% CI: −2.30; −0.14, *p* = 0.027) in the multicomponent online group compared to the control group (Table 3).

**Table 3:** Effect of multicomponent online intervention on anthropometric measures, body composition, muscle strength, physical performance, and resting energy expenditure in individuals in the late postoperative period of metabolic bariatric surgery

Variables	Multicomponent online group		Control group		β^1^ (95% CI)	*p* value^2^
	Baseline	After 12 weeks	Baseline	After 12 weeks		
	Mean (95% CI)	Mean (95% CI)	Mean (95% CI)	Mean (95% CI)		
BW (kg)	80.7 (77.0; 84.3) ^aA^	79.9 (76.2; 83.5) ^aA^	78.5 (74.4; 82.6) ^aA^	79.2 (74.9; 82.6) ^aA^	−1.52 (−3.09; 0.05)	0.058
FFM (kg)	50.6 (48.7; 52.4) ^aA^	50.8 (48.9; 52.7) ^aA^	48.3 (46.3; 50.4) ^aA^	48.6 (46.3; 50.9) ^aA^	−0.07 (−1.17; 1.02)	0.895
SMM (kg)	27.7 (26.6; 28.8) ^aA^	27.9 (26.8; 29.0) ^aA^	26.4 (25.2; 27.9) ^aA^	26.5 (25.2; 27.9) ^aA^	−0.006 (−0.63; 0.62)	0.985
FM (kg)	30.1 (27.6; 32.6) ^aA^	29.5 (27.1; 32.0) ^aA^	30.0 (27.2; 32.9) ^aA^	31.1 (27.8; 34.4) ^aA^	−1.60 (−3.63; 0.43)	0.123
BF (%)	36.4 (34.7; 38.1) ^aA^	35.9 (34.1; 37.6) ^aA^	37.4 (35.4; 39.3) ^aA^	38.0 (35.9; 40.2) ^aA^	−1.17 (−2.77; 0.43)	0.153
FFM/BW x 100 (%)	63.6 (61.9; 65.3) ^aA^	64.5 (62.8; 66.3) ^aB^	62.6 (60.4; 64.8) ^aA^	62.2 (59.9; 64.6) ^aA^	1.28 (−0.79; 3.35)	0.226
SMM/BW x 100 (%)	34.8 (33.8; 35.7) ^aA^	35.3 (34.4; 36.3) ^aB^	34.1 (32.9; 35.3) ^aA^	33.9 (32.6; 35.2) ^aA^	0.77 (−0.40; 1.94)	0.198
FM/BW x 100 (%)	36.4 (34.7; 38.1) ^aA^	36.0 (34.0; 38.1) ^aA^	37.3 (35.3; 39.3) ^aA^	38.6 (35.3; 41.9) ^aA^	−1.57 (−4.78; 1.64)	0.337
PhA (º)	5.1 (5.0; 5.2) ^aA^	5.2 (5.1; 5.4) ^bB^	5.0 (4.9; 5.2) ^aA^	4.9 (4.8; 5.1) ^aA^	0.15 (0.02; 0.29)	**0.025**
STS_5r (s)	10.2 (9.8; 10.7) ^aA^	8.7 (8.0; 9.4) ^aB^	9.9 (9.4; 10.5) ^aA^	9.6 (8.8; 10.4) ^aA^	−1.22 (−2.30; −0.14)	**0.027**
HGS/BW (kgf/kg)	0.37 (0.34; 0.40) ^aA^	0.41 (0.38; 0.43) ^aB^	0.39 (0.37; 0.41) ^aA^	0.39 (0.37; 0.41) ^aA^	0.03 (0.001; 0.06)	**0.043**
TUG (s)	6.1 (6.0; 6.3) ^aA^	6.2 (6.1; 6.5) ^aA^	6.3 (6.0; 6.5) ^aA^	6.5 (6.3; 6.8) ^aA^	−0.07 (−0.45; 0.32)	0.731
10 MWT (s)	4.8 (4.5; 5.0) ^aA^	4.9 (4.5; 5.4) ^aA^	4.8 (4.6; 5.0) ^aA^	4.6 (4.4; 4.9) ^aA^	0.36 (−0.20; 0.92)	0.203
REE (kcal/day)	1550 (1502; 1599) ^aA^	1537 (1485; 1587) ^aA^	1508 (1450; 1566) ^aA^	1530 (1459; 1601) ^aA^	−35.49 (−106.67; 35.69)	0.328
REE/BW (kcal/kg)	19.6 (19.1; 20.1) ^aA^	19.7 (19.0; 20.4) ^aA^	19.6 (18.9; 20.4) ^aA^	19.6 (18.8; 20.4) ^aA^	0.16 (−0.78; 1.10)	0.738
REE/FFM (kcal/kg)	30.9 (30.2; 31.6) ^aA^	30.5 (29.6; 31.4) ^aA^	31.5 (30.6; 32.4) ^aA^	31.5 (30.4; 32.6) ^aA^	−0.39 (−1.82; 1.05)	0.599
Respiratory quotient	0.82 (0.82; 0.83) ^aA^	0.84 (0.83; 0.85) ^aB^	0.83 (0.82; 0.84) ^aA^	0.83 (0.82; 0.85) ^aA^	0.01 (−0.007; 0.03)	0.188

^1^ Unstandardized beta between group comparison at the post-intervention time. ^2^ Generalized estimating equations adjusted for the baseline. CI confidence interval; BW body weight; FFM fat-free mass; SMM skeletal muscle mass; FM fat mass; BF body fat; BW body weight; 10 MWT 10-Meter Walk Test; PhA phase angle; STS_5r five repetitions sit-to-stand test; HGS dominant handgrip strength; TUG Timed-up-and-go test; REE resting energy expenditure

Lowercase letters indicate between-group comparisons at each time point. Uppercase letters indicate within-group comparisons over time

At baseline, the intervention group presented a higher prevalence of sarcopenia risk compared with the control group, indicating an initial disadvantage between groups. There was no within-group change in sarcopenia risk in the control group after the 12-week study period. However, the multicomponent online group showed a marginally significant reduction in sarcopenia risk (*p* = 0.084). After 12 weeks, the intervention group demonstrated a lower likelihood of sarcopenia risk compared with the control group (OR = 0.41; 95% CI: 0.19; 0.87, *p* = 0.020). No differences were observed in obesity classification odds either between or within groups after the study (Table 4).

**Table 4:** Distribution of sarcopenia risk and obesity classification in the study groups at baseline and after 12 weeks of intervention, and the odds ratio for modification these conditions following the proposed intervention

Variables	Multicomponent online group		Control group		OR (95% CI)	*p* value^1^
	Baseline	After 12 weeks	Baseline	After 12 weeks		
	%	%	%	%		
Sarcopenia risk^1^	66.7^bA^	59.5^bB^	56.7^aA^	63.9^aA^	0.41 (0.19; 0.87)	**0.020**
Obesity^2^	63.3^aA^	55.0^aA^	64.4^aA^	68.8^aA^	0.53 (0.25; 1.13)	0.100

lowercase letters indicate between-group comparisons at each time point. Uppercase letters indicate within-group comparisons over time. ^1^ Generalized estimating equations adjusted for the baseline

## Discussion

The results of this clinical trial demonstrate the clinical utility of a remote, multicomponent strategy—integrating nutritional counseling, behavioral techniques, and supervised exercise of a multicomponent online intervention combining nutritional counseling with a behavioral strategy, and supervised exercise training, all monitored by healthcare professionals, during the late postoperative period of MBS. The salient findings indicated that the intervention effectively improved sarcopenia-related traits, highlighting its potential as a clinically relevant approach to preserve muscle health in this population. Collectively, these findings support the importance of a multidisciplinary, professionally guided strategy for addressing postoperative complications and demonstrate that online delivery can serve as a feasible and scalable alternative to traditional in-person interventions.

No standardized face-to-face or online protocol exists for bariatric surgery follow-up, with variations in intervention type, volume, and duration contributing to heterogeneity in systematic reviews [19, 46–48]. This study implemented a multicomponent online intervention in the late postoperative period, addressing barriers of in-person care such as transportation [49] and time constraints [50]. The intervention, combining dietary improvement and supervised online exercise, effectively increased strength and decreased sarcopenia risk, independent of BMI or obesity classification changes, indicating benefits beyond body composition.

More than half of the participants had experienced recurrent weight gain prior to enrollment—a phenomenon commonly observed after the second postoperative year [51]. At baseline, participants were not engaged in structured nutritional monitoring or physical activity programs—factors that likely contributed to this weight gain and may increase the risk of sarcopenia and associated health complications [52]. Notably, during the intervention, body weight in the multicomponent online group remained stable, suggesting that the program effectively counteracted further weight gain—a clinically meaningful outcome in this population.

In the multicomponent online group, attendance was higher for the weekly online nutritional counseling sessions than for the thrice-weekly exercise sessions, likely due to the lower frequency facilitating participation. Overall adherence was suboptimal according to the commonly used ≥ 70% threshold in intervention studies, with a 62.6% attendance rate, but this level of participation may reflect engagement patterns closer to real-world conditions. Importantly, even with suboptimal adherence, the intervention was associated with improvements in strength and reductions in the risk of sarcopenia, suggesting that the program may still provide meaningful benefits despite less-than-ideal attendance. Higher adherence was associated with older age and greater educational level, consistent with previous findings [53, 54], possibly reflecting better understanding of health benefits and more available time to participate in the proposed multicomponent online intervention.

Our findings related to muscle strength align with those of previous studies using similar exercise protocols. Baillot et al. [55] conducted an online intervention in women awaiting bariatric surgery, while Lamarca et al. [56] implemented an in-person protocol in late postoperative patients. Both reported significant improvements in muscle strength. Increased muscular strength correlates with better performance in daily activities among individuals with obesity [57, 58] and may reduce fall risk [59]. Such gains are especially relevant in the late postoperative period of MBS, which is often marked by recurrent weight gain [51] and a consequent functional decline [60]. The substantial strength gains observed in the intervention group, particularly among previously untrained individuals, are consistent with the improvements typically seen within the first 2–3 months of training, which are primarily attributed to neural mechanisms such as enhanced motor unit recruitment and improved neuromuscular activation [61, 62].

Given the observed improvements in muscle strength, preserving muscle mass remains clinically relevant, particularly due to the risk of FFM and skeletal muscle loss within the first year post-bariatric surgery, which contributes to sarcopenia [63] and its associated adverse outcomes [64, 65]. Routine monitoring of body composition, especially PhA from BIA [66], provides insight into muscle quality [63] and sarcopenia risk [28], as lower PhA values are associated with reduced muscle mass and function [67]. Despite the growing recognition of phase angle as a promising screening tool for sarcopenia [28], the establishment and standardization of cut-off points still require further multicenter studies across diverse populations, including individuals in the postoperative period following MBS. The reduced sarcopenia risk observed in the multicomponent online group underscores the potential of online multicomponent strategies to mitigate muscle mass decline and improve strength outcomes. Although baseline differences in sarcopenia risk existed, GEE models adjusted for them; so, the reported β coefficients reflect intervention effects.

FFM is a major determinant of REE, mainly due to metabolically active organs [68, 69], and to a lesser extent SMM [70]. This study identified no significant REE changes, likely because the intervention duration or intensity were insufficient to produce measurable differences in FFM or SMM between groups, unlike longer training protocols reported elsewhere [71].

Despite the higher fasting glucose observed in the multicomponent online group, it is important to note that this increase was minor and remained within the normal clinical range (Table 2). In the late postoperative period of bariatric surgery, the pancreas may operates in a state of extreme metabolic adaptation or functional exhaustion [72], which accounts for the sub-reference baseline HOMA-beta values. Resistance exercise enhances insulin sensitivity and the efficiency of muscular glucose uptake through GLUT4 translocation [73]. Consequently, there is a decreased secretory demand on pancreatic beta cells (the insulin-sparing effect), leading to a further decline in HOMA-beta (Table 2). In this context, the slight increase in fasting plasma glucose, coupled with stable HbA1c levels, reflects the achievement of a new glycemic homeostasis set point, sustained by improved peripheral efficiency rather than compensatory hyperinsulinemia.

Consistently, exercise has been shown to reduce systemic inflammation, as indicated by lower levels of C-reactive protein [74], as can also be observed in the intervention group after the study (Table 2). Exercise also may have increased serum creatinine without impacting renal function [75].

This study’s strengths include intimate supervision by trained professionals and flexible scheduling to enhance adherence. It also provides valuable evidence on online multicomponent interventions in the long-term postoperative bariatric population, where data remain scarce. However, a high dropout rate occurred (~ 57%), with specific reasons detailed in the flow diagram (Fig. 1). In both groups, the primary barriers were personal or family issues and time constraints, followed by health-related problems and loss of interest. Such attrition highlights the significant challenges of sustaining engagement in intensive lifestyle interventions within this population. Suboptimal adherence, which is common in lifestyle-based clinical trials and real-world settings, may limit the generalizability of the findings, particularly given the predominantly female, single-center sample. The high dropout rate and suboptimal adherence may have influenced the magnitude of the results. Furthermore, weight regain was assessed retrospectively based on the lowest weight reported by participants, which is subject to recall bias. While time since surgery was considered, its strong collinearity with weight regain limited its inclusion as an independent covariate in the analysis. Future implementations of this intervention may benefit from strategies aimed at improving adherence, such as greater scheduling flexibility, enhanced behavioral support, and additional engagement strategies. Nevertheless, an intention-to-treat analysis was applied to preserve initial randomization and minimize bias from differential dropout groups, preventing overestimation of treatment effects. Additionally, the absence of an on-site, supervised face-to-face intervention group limits direct comparisons between online and in-person exercise-based programs. Likewise, the lack of low-intensity and asynchronous online comparators restricts inferences regarding intervention intensity and delivery format. Future studies are needed to compare different intervention approaches and to improve external validity.

## Conclusion

This study demonstrates that a 12-week supervised online multicomponent intervention, integrating nutritional counseling and structured exercise, improves muscle strength, PhA, β-cell function, and reduces C-reactive protein levels and sarcopenia risk in individuals in the late postoperative period of MBS. Importantly, these benefits occurred independently of changes in body weight or total energy expenditure, highlighting the importance of evaluating a broader range of physiological parameters beyond weight alone. Focusing solely on weight may prevent patients from recognizing other positive metabolic and behavioral adaptations that are essential for long-term health. Such adaptations can serve as drivers for greater adherence to treatment and facilitate the achievement of primary goals. Given that weight loss through behavioral interventions may require extended periods, recognizing these intermediate benefits is crucial for maintaining motivation and engagement. Thus, these findings demonstrate that a supervised, remote multicomponent intervention is a feasible and effective strategy for improving muscle health and mitigating sarcopenia risks in long-term bariatric care. However, extended studies with greater adherence are required to confirm these findings and explore the long-term impact on metabolic health and quality of life.

## Supplementary Information

Below is the link to the electronic supplementary material.

Supplementary Material 1

## Data Availability

No datasets were generated or analysed during the current study.
