# Supplementary material for: Multicomponent Online Intervention Improves Sarcopenia-Related Traits Following Long-Term Metabolic Bariatric Surgery: A Randomized Clinical Trial
Source: Obes Surg. 2026 May 22;36(7):3632–45. doi: 10.1007/s11695-026-08742-x (PMC13323218; doi:10.1007/s11695-026-08742-x)
Supplement: Supplementary file 1 — Supplementary Material 1 [file 11695_2026_8742_MOESM1_ESM.docx]

**Multicomponent Online Intervention Improves Sarcopenia-Related Traits Following Long-Term Metabolic Bariatric Surgery: A Randomized Clinical Trial**

Table of Content

[Table 1 Baseline Demographic and Clinical Characteristics of Participants in the Late Postoperative Period of Metabolic Bariatric Surgery, stratified by dropout and attended sample. 1](#_Toc225857318)

[Table 2 Behavioral-Based Nutritional Counseling Intervention Details 2](#_Toc225857320)

[Table 3 Structure and Objectives of Synchronous Nutritional Sessions 3](#_Toc225857321)

[Table 4 Supervised Remote Physical Exercise Program Details 8](#_Toc225857322)

[Table 5 Circuits of Remote Physical Exercise Program 9](#_Toc225857323)

[Schematic illustration of the aerobic and resistance exercises proposed in the study training protocol 10](#_Toc225857324)

[References 15](#_Toc225857325)

#

# Demographic and clinical characteristics between participants who completed the study and those who withdrew

## Table 1 Baseline Demographic and Clinical Characteristics of Participants in the Late Postoperative Period of Metabolic Bariatric Surgery, stratified by dropout and attended sample.

| Variables | Discontinued the study (n = 79) | Completed the study (n = 60) | *p* value |
| --- | --- | --- | --- |
| Female [n (%)] | 73 (92.4) | 53 (88.3) | 0.414^1^ |
| Age (years) | 41.0 ± 8.0 | 39.1 ± 7.3 | 0.303^2^ |
| Education level (years of study) | 14.6 ± 2.8 | 14.5 ± 2.7 | 0.850^2^ |
| RYGB surgical procedure [n (%)] | 72 (91.1) | 59 (98.3) | 0.138^3^ |
| Years after surgery (years) | 4.0 ± 1.7 | 3.8 ± 1.5 | 0.734^2^ |
| Preoperative body mass index (kg/m^2^) | 41.7 ± 5.5 | 42.7 ± 5.8 | 0.330^2^ |
| Current body mass index (kg/m^2^) | 29.0 ± 5.2 | 29.9 ± 4.8 | 0.132^2^ |
| Excess weight loss (%) | 80.4 ± 25.2 | 75.3 ± 25.4 | 0.246^4^ |
| Total weight loss (%) | 38.6 ± 7.6 | 38.4 ± 6.5 | 0.851^4^ |
| Recurrent weight gain^5^ [n (%)] | 46 (58.2) | 39 (65.0) | 0.593^1^ |
| Mean of recurrent weight gain (n = 85) (%) | 13.9 ± 9.5 | 14.3 ± 9.3 | 0.856^2^ |
| Mean of usual energy intake (kcal/day) | 1755 ± 487 | 1859 ± 477 | 0.111^2^ |
| Mean of usual protein intake (g/day) | 84.1 ± 22.4 | 87.2 ± 22.4 | 0.372^2^ |
| Mean of MET-h/day | 1.39 ± 0.17 | 1.36 ± 0.18 | 0.444^2^ |

^1^ Chi-square test; ^2^ Mann–Whitney U test; ^3^ Fisher´s exact test ^4^ Student’s t test for independent samples; ^5^ Recurrent weight gain when > 10% of the lowest weight obtained in the postoperative period. MET-hour/day metabolic equivalent of task-hour/day; RYGB Roux-en-Y gastric bypass.

# Multicomponent Intervention Description

## Table 2 Behavioral-Based Nutritional Counseling Intervention Details

| **Component** | **Description** |
| --- | --- |
| Approach | Group-based behavioral nutritional counseling program with a behavioral approach, based on the Brazilian Cardioprotective Diet [1]. |
| Frequency and Duration | Weekly sessions, 60 minutes each, for 12 consecutive weeks. |
| Delivery Mode | Group-based synchronous online meetings via video conferencing platform (supervised in real-time by one dietitian per session); asynchronous video lectures (13 videos, ~20 minutes each). |
| Communication Platform | Smartphone messaging app for sending materials and facilitate communication between participants and the research team. |
| Dietitian-to-Participant Ratio | 1:8 (average) |
| Supervision | By a trained dietitian, who led the session, addressed any questions related to the topic covered, and ensured that all participants engaged in the group discussions. |
| Video Topics (asynchronous content) | 1. The importance of restorative sleep, adequate hydration, and strategies to improve water intake and sleep quality.  2. Types of hunger (physiological, hedonic, social, and emotional), how to recognize them, and strategies to manage each one effectively.  3. How emotions and the environment may influence the perception of hunger and satiety.  4. The role of mindfulness eating in managing emotional hunger.  5. Physiological hunger, how to identify it, and how mindfulness eating can enhance satiety.  6. Health consequences of restrictive diets and strategies for fostering a healthy relationship with food.  7. Body image and the importance of self-acceptance.  8. Understanding how foods are classified based on their level of processing, according to the Brazilian Dietary Guidelines [1], and how to apply this classification in practice by reading food labels.  9. Classification of foods according to the Brazilian Cardioprotective Diet [2].  10.Emphasis on consuming cardioprotective, nutrient-dense foods and limiting high-calorie foods rich in unhealthy fats, given their link to increased cardiovascular risk.  11. Applying food classification principles based on the Brazilian Cardioprotective Diet [2].  12. Healthy substitutes for ultra-processed foods mistakenly considered healthy, according to the Brazilian Cardioprotective Diet.  13. Understanding how to identify salt and sugar in foods and apply strategies to reduce their daily consumption. |
| Practical Activities | Weekly activities based on video topics to reinforce behavioral change (e.g., mindful eating practices, food label reading, hunger/satiety journaling). |
| Support Tools | Video library (an online video platform links), interactive group discussions conducted through live online meetings with a trained dietitian, personalized weekly challenges. |

## Table 3 Structure and Objectives of Synchronous Nutritional Sessions

| **Session** | **Objective** | **Main content** | **Home Assignment** | **Activities performed** |
| --- | --- | --- | --- | --- |
| 1- Sleep and Hydration | To present the project and learn about the importance of sleep and hydration; to explore strategies to improve water intake and sleep quality | - Brief presentation of the project team and explanation of the structure of the nutrition sessions.  - Review of the video content on the role of sleep and hydration in health, hunger regulation, and body weight control.  - Discussion of practical strategies to improve sleep hygiene and water consumption. | - Establish fixed times for waking up and going to bed.  - Dim household lights in the evening.  - Avoid distractions for at least one hour before bedtime. | - Ice-breaker activity: participants introduced themselves and answered “How did you sleep last night?”  - Clarification of doubts.  - Group sharing of personal strategies to enhance sleep and hydration.  - Reinforcement of the home assignment proposed in the video (*https://youtu.be/NUK0AGYnaaw*) |
| 2- Types of Hunger | To understand the different types of hunger, how to recognize them, and learn strategies to manage each one effectively. | - Review of hunger types: physiological hunger, craving, social hunger, and emotional hunger.  - Guidance on how to identify each type and apply appropriate coping strategies. | - Record hunger/fullness levels before meals.  - Identify the type of hunger experienced.  - Reinforce the message that one should not feel guilty, but rather allow themselves to eat consciously and enjoy the food, returning to mindful eating practices. | - Guided discussion based on the educational video (*https://youtu.be/OSHxGQ-i5Qc*) - Clarification of doubts.  - “Which hunger am I?” activity to help participants identify the type of hunger being described.  - Sharing and reinforcement of the home assignment proposed in the video. |
| 3 - Influence of Emotions and Environment on Hunger and Satiety | To understand the different types of hunger and learn strategies to manage each one. | - Review of the video content on hunger and satiety.  - Explanation of the importance of recognizing hunger and satiety cues.  - Discussion on how eating in a calm environment can enhance these perceptions.  - Explanation of the emotional impact on eating behavior. | - Select two meals during the week to be eaten in a calm setting.  - Record the primary emotion experienced during each meal.  - Note hunger and satiety levels before and after the meal.  - Indicate whether the meal was eaten alone or with company. | - Sharing and reinforcement of the home assignment proposed in the video (*https://youtu.be/AnQU3kziyLo*) - Group activity to collaboratively develop strategies for managing emotions without turning to food.  - Presentation of practical techniques for distraction, emotional support, self-care, and emotional regulation without using food as a coping mechanism. |
| 4 – Emotional Hunger and Mindful Eating | To learn about mindful eating and identify which foods satisfy emotional/psychological hunger. | - Review of the video content. - Explanation of mindful eating: bringing full attention to the present moment while eating, without guilt or judgment.  - Strategies to address emotional hunger:  1) Reconnect with hunger and satiety cues;  2) Prioritize physical hunger—plan main meals to avoid emotional hunger later;  3) Let go of the idea of “forbidden foods”;  4) Avoid labeling foods as “good” or “bad”;  5) Identify foods that genuinely satisfy emotional hunger. | - Choose a food that typically triggers guilt or self-judgment.  - Plan a moment to eat it mindfully.  - Record thoughts and feelings before and after the experience. | - Ice-breaker activity: participants introduced themselves and answered “What have you been craving lately?”  - Group discussion on barriers to practicing mindful eating and shared strategies to overcome them.  - Reinforcement of the home assignment proposed in the video (*https://youtu.be/p-WfPrbtY30)* |
| 5- Recognizing Physiological Hunger and Satiety | To learn how to identify physical signs of physiological hunger and satiety. | - Review of the video content on physiological hunger and satiety: definitions, how to identify them, and the role of mindful eating in managing food intake, particularly of processed and ultra-processed foods.  - Discussion on how tuning into hunger and satiety signals supports healthier eating patterns. | - Record the time and place of lunch meals.  - Note the main emotion associated with the meal.  - Indicate the level of hunger and satiety before and after the meal. | - Clarification of questions and doubts.  - Interactive activity “Recognizing Your Hunger”: participants practiced identifying their hunger and satiety levels using a 1–10 scale.  - Reinforcement of the home assignment proposed in the video (*https://youtu.be/_ghYEFm1SIk?si=dsvfiuuKXbfojbWX*) |
| 6- Restrictive Diets and Making Peace with Food | To share personal experiences with restrictive diets and discuss strategies to make peace with food. | - Review of the video’s key messages: the long-term consequences of restrictive diets on health; strategies to make peace with food; differences between intuitive eating and diet mentality.  - Emphasis on the importance of prioritizing intuitive eating over diet thinking. | - Construct a personal timeline highlighting weight history and restrictive diets followed throughout life. | - Clarification of questions and doubts.  - Group discussion based on reflective questions: “What do you believe about diets and weight loss?”, “Where did those beliefs come from?”, “What do you think will happen if you lose weight?”, “Do you put life plans on hold until you lose weight?”, “What would it be like to start exploring those things now, even without losing weight?”.  - Reinforcement of the home assignment proposed in the video (*https://youtu.be/dXT2ytoXOTA*) |
| 7- Body Image and Self-Acceptance | To discuss body image and encourage self-acceptance. | - Explanation of the concept of body image and the importance of accepting oneself.  - Discussion of factors that influence body image: social comparison, body type diversity, social media influence, and the pursuit of perfection.  - Emphasis on striving to become the best version of oneself rather than an idealized version. | - List three personal characteristics that you like about yourself and three obstacles that prevent you from liking yourself fully. | - Presentation of real-life examples and strategies to promote a healthier body image.  - Group reflection and discussion about home assignment proposed in the video (*https://youtu.be/M9IzuYaUs48)* |
| 8- NOVA Food Classification and Label Reading | To introduce the NOVA food classification system based on the Brazilian Dietary Guidelines [1], including how to classify foods and read labels. | - Recap of the recorded video content on NOVA food classification (unprocessed/minimally processed, processed, and ultra-processed foods).  - Discussion on food labeling and how to identify food groups. | - Define a weekly fruit consumption goal.  - Perform the "fruit meditation" exercise based on the step-by-step guide in the shared video (*https://youtu.be/TTc7kC1WquI*). | - “Bingo” activity to reinforce the NOVA food classification through practical examples.  - Group reflection and discussion. About home assignment proposed in the video (*https://youtu.be/9cqHvA5iQME)* |
| 9- Brazilian Cardioprotective Diet: Food Classification by Color Groups | To reinforce knowledge on the Brazilian Cardioprotective Diet [2] using the color-coded food classification inspired by the Brazilian flag. | - Review of key points from the recorded session: classification of foods based on the Brazilian Cardioprotective Diet using the green, yellow, and blue colors of the national flag.  - Explanation of energy density vs. nutritional density. | - Choose one food from the green group (low energy density) that was not habitually consumed and include it in meals during the week.  - Apply the mindful eating practice with the selected food.  - Frequency and portion goals were defined individually. | - Interactive activity using food images: participants helped classify the foods by placing them on an image of the Brazilian flag, according to the corresponding color group.  - Participants were called by name to encourage engagement.  - Group reflection and discussion. About home assignment proposed in the video (*https://youtu.be/BpyhiLIoItk*) |
| 10 - Cardioprotective Nutrients and Food Groups | To explore the nutrients present in the food groups of the Brazilian Cardioprotective Diet [2] and their physiological roles. | - Review of the food groups within the Cardioprotective Diet.  - Explanation of the importance of cardioprotective nutrients (e.g., antioxidants, vitamins, minerals, fiber) found in fruits, vegetables, legumes, and dairy products.  - Identification of foods from the red group that should be limited (those high in trans and saturated fats), emphasizing planning rather than restriction. | - Prepare a lunch or dinner plate based on the principles of the Cardioprotective Diet.  - Reflect on whether it differed from usual meals and whether all three groups were included. | - Guided discussion about participants’ experiences preparing meals according to the cardioprotective dietary pattern: How was the meal assembly? Was it different from their usual plate? Were they able to include foods from all three groups?  - Interactive activity: participants assembled a lunch plate using on-screen food items, categorizing them by the color-coded Cardioprotective Diet groups (green, yellow, blue).  - Group reflection and discussion. About home assignment proposed in the video (*https://youtu.be/OIeSW0SNoN0)* |
| 11 - Practical Application of Food Classification based on the Brazilian Cardioprotective Diet (DiCABr) | To apply the classification of foods into groups of the Brazilian Cardioprotective Diet [2] in practice. | - Recap of the previous session: classification of foods according to DiCABr, NOVA food classification, and label reading.  - Practical demonstration on how to classify food items into the appropriate DiCABr groups.  - Presentation of foods belonging to the same general category (e.g., dairy, bakery) but differing in DiCABr group classification based on nutritional profile and processing. | - Read the labels of all food products commonly consumed at home over one day (e.g., bread, yogurt, cheese, milk, cookies).  - Classify each item according to the DiCABr groups using both NOVA and Cardioprotective Diet criteria. | - Group discussion and classification exercises using real food packaging or visual examples to reinforce understanding of differences among similar foods according to DiCABr.  - Group reflection and discussion. About home assignment proposed in the video (*https://youtu.be/L2kC0bKZVtw)* |
| 12 - Food Classification and Healthy Substitutions (DiCABr) | To reinforce food classification according to the Brazilian Cardioprotective Diet (DiCABr) and promote healthier food substitutions through interactive strategies. | - Recap of the recorded session: classification of supermarket foods using the DiCABr framework; examples of processed and ultra-processed foods perceived as healthy (e.g., flavored yogurts with additives, turkey breast) and corresponding healthier alternatives.  - Clarification of doubts regarding food classification and substitutions. | - Observe which of the ultra-processed foods shown in the video (<https://youtu.be/1SqoQiQDqZo>) are part of the participant’s usual diet.  - Attempt to replace them with healthier options throughout the week. | - Interactive "shopping cart" game: participants selected four foods from slides and scored them based on DiCABr classification.  - Scoring system: Green group = 10 pts/item; Yellow group = 8 pts (or 9 pts for whole grains, nuts, and olive oil); Blue group = 6 pts; Red group = −1 pt/item.  - Group discussion on score outcomes, rationale behind point values, and classification validation.  - Guided questions: Why does the Green group score highest? Why do olive oil, nuts, and brown rice score higher within the Yellow group? Why do Red group items result in point deduction? |
| 13 - Sugar and Salt in Processed Foods | To understand how to identify the presence of added sugar and salt in foods and apply strategies to reduce their intake. | - Review of recorded videos (Parts 1 - <https://youtu.be/60_birDuILU> and 2 - (https://www.youtube.com/watch?v=9N8m1HRbpEc): health risks associated with excessive sugar and salt consumption.  - Explanation of how to identify high sugar/sodium content on food labels.  - Discussion of the importance of reducing intake to prevent cardiovascular and metabolic diseases. | - Select a sweet or savory item and replace it with a healthier alternative.  - Mindfully enjoy the substitution, documenting the experience, taste perception, and any encountered difficulties. | - Clarification of participants' questions related to sugar/salt identification and health impact.  - Group sharing of the home assignment experiences: substitutions made, challenges encountered, and personal reflections.  - Interactive roundtable: participants exchanged strategies to reduce sugar and salt intake in daily life. |

## Table 4 Supervised Remote Physical Exercise Program Details

| **Component** | **Description** |
| --- | --- |
| Approach | Group-based supervised remote physical exercise program, designed and adapted in accordance with the American College of Sports Medicine (ACSM) guidelines for individuals with obesity [3]. |
| Frequency and Duration | 3 sessions per week, on non-consecutive days, ~60 minutes each, for 12 consecutive weeks. |
| Delivery Mode | Group-based synchronous online meetings via video conferencing platform (supervised in real-time by one certified Physical Education professional per session). |
| Communication Platform | Smartphone messaging app for sending materials (instructional videos with demonstrations of all exercises) and facilitate communication between participants and the research team. |
| Instructor-to-Participant Ratio | 1:8 (average) |
| Supervision | By a qualified physical education professional, who conducted the sessions, addressed any questions regarding exercise movements, provided corrections related to execution, and proposed injury prevention strategies. |
| Exercise Objectives | Improve cardiorespiratory fitness and muscular strength |
| Session Structure | 5-minute warm-up → 3 circuits (resistance + aerobic training) → 5-minute cool-down with stretching and breathing |
| Familiarization Phase | 3 sessions prior to program start to ensure proper technique |
| Load Monitoring Tool | OMNI-Resistance Exercise Scale (OMNI-RES) [4] |
| Progressive Load Scheme | Weeks 1–4: OMNI-RES = 6 ("somewhat hard"); Weeks 5–8: OMNI-RES = 7; Weeks 9–12: OMNI-RES = 8 ("hard") |
| Safety Measures | Participants monitored in real time; advised to stop upon signs of pain, discomfort, or dizziness |
| Activity Restrictions | Participants instructed not to engage in other structured physical training during the intervention period |
| Equipment Provided | Dumbbells (2 kg, 3 kg, 5 kg, 8 kg), exercise mat |
| Resistance Component | 3 circuits with 3 exercises each; 2 sets of 10–12 reps; 60 seconds rest between circuits |
| Aerobic Component | 5 minutes of aerobic exercise preceding each circuit (e.g., marching in place, jogging, jumping jacks) |

## Table 5 Circuits of Remote Physical Exercise Program

| **Aerobic (before each circuit) ^1^** | **Circuit 1 ^2^** | **Circuit 2 ^2^** | **Circuit 3 ^2^** |
| --- | --- | --- | --- |
| ***5 min aerobic before each circuit*** (total aerobic time per session: 15 min) | *Unilateral bent-over row*  Intensity progression:  - Load increase ^3^ | *Supine chest fly*  Intensity progression:  -Load increase ^3^ | *Triceps kickback (“donkey kick”)*  Intensity progression:  - Load increase ^3^ |
| **Intensity progression:**  – Step forward and backward (level 1)  – Marching in place (level 2)  – Stationary running (level 3)  – Jumping jacks (level 4) | *Squat*  Intensity progression:  - With chair (level 1),  - Without chair (level 2),  - With load (level 3) | *Calf raises*  Intensity progression:  - Bilateral (level 1),  - Unilateral (level 2) | *Bicep’s curl*  Intensity progression:  - Load increase ^3^ |
|  | *Standing shoulder press*  Intensity progression:  -Load increase ^3^ | *Plank on mat*  Intensity progression by duration:  - 15″ (level 1),  - 30″ (level 2),  - 45″ (level 3),  - 60″ (level 4) | *Hip raise on mat*  Intensity progression:  - Bilateral (level 1),  - Perpendicular arms (level 2),  - Unilateral (level 3) |

The table describes the components of each session, including aerobic exercise^1^, and resistance training^2^. Training load^3^ was monitored using the OMNI-Resistance Exercise Scale (OMNI-RES) and progressively adjusted throughout the intervention according to participants’ perceived exertion.

## Schematic illustration of the aerobic and resistance exercises proposed in the study training protocol

1. Aerobic exercises performed prior to each circuit


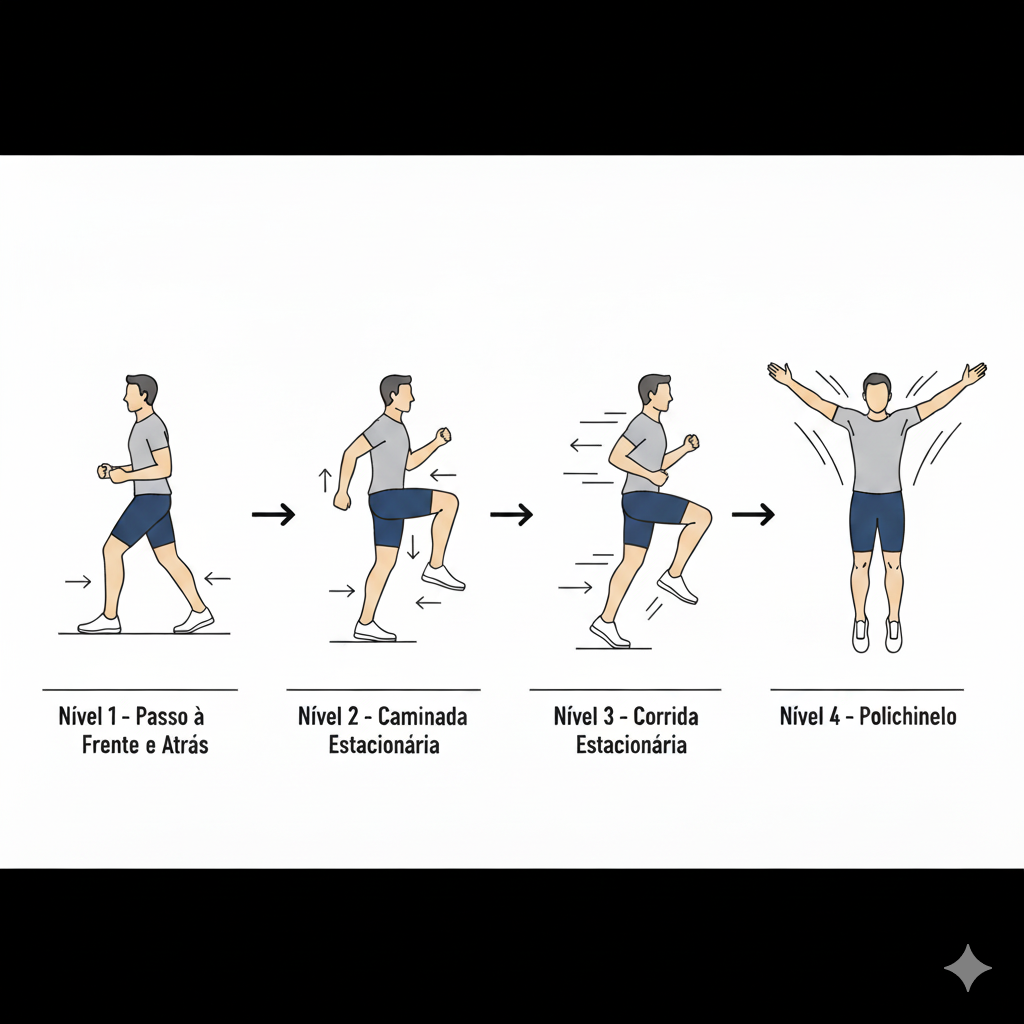


Level 1 - Step forward Level 2 - Marching Level 3 - Stationary Level 4 - Jumping

and backward in place running jacks

1. Circuit 1 - Unilateral bent-over row (Intensity progression: load increase)


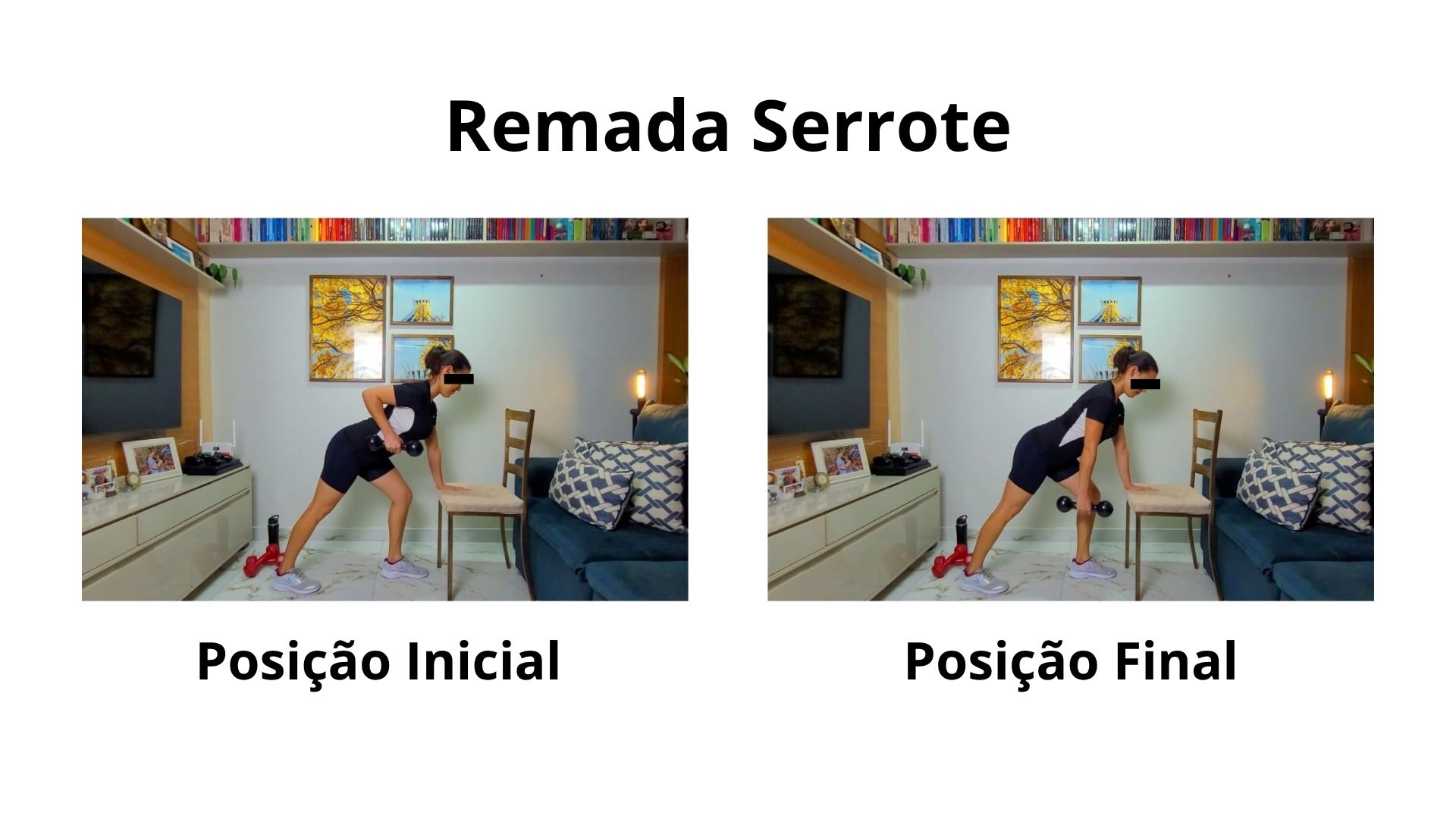


Initial position Final position

1. Circuit 1 - Squat (level 1: with chair)


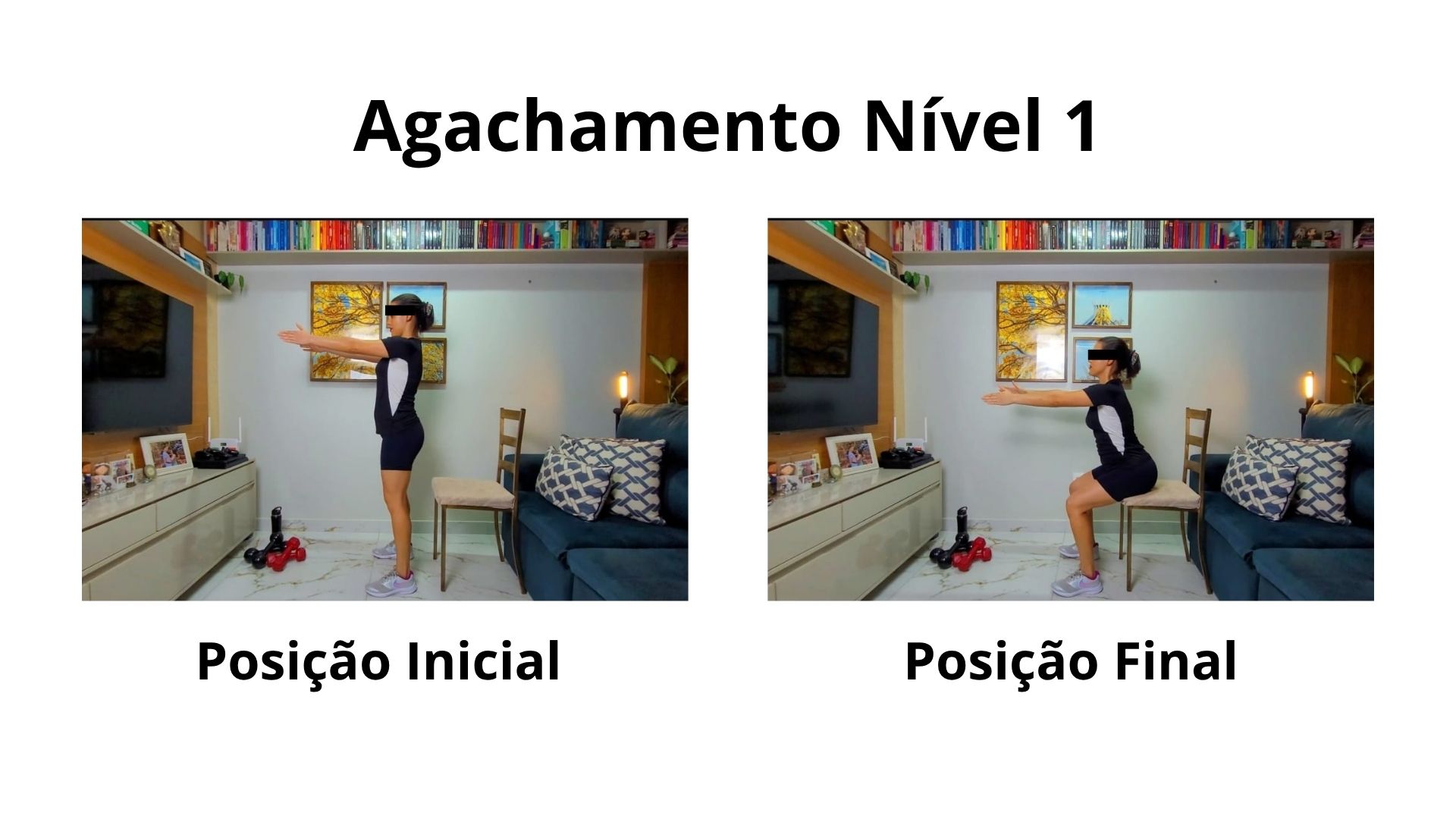


Initial position Final position

1. Circuit 1 - Squat (level 2: without chair)


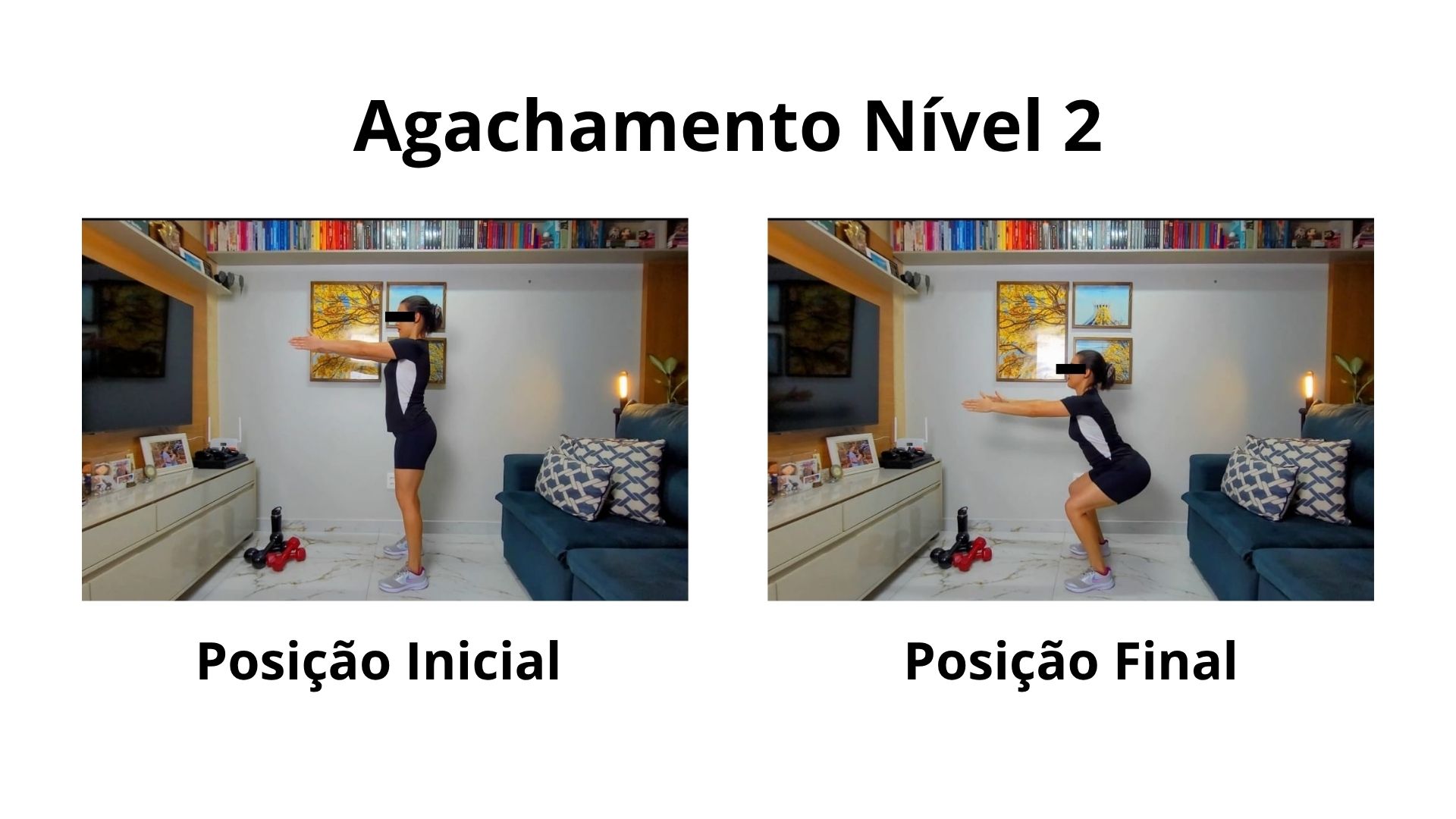


Initial position Final position

1. Circuit 1 - Squat (level 3: with load)


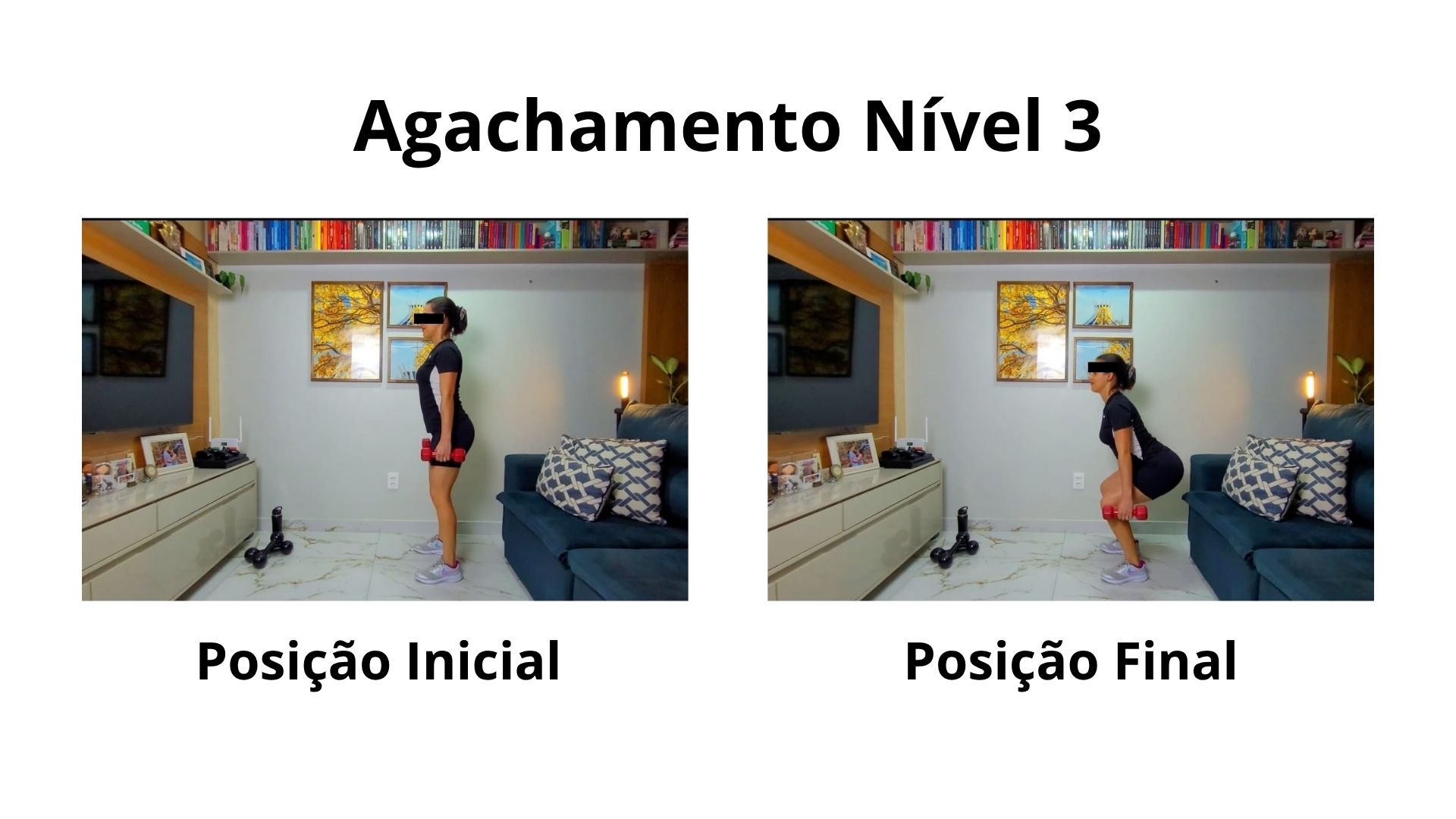


Initial position Final position

1. Circuit 1 - Standing shoulder press (Intensity progression: load increase)


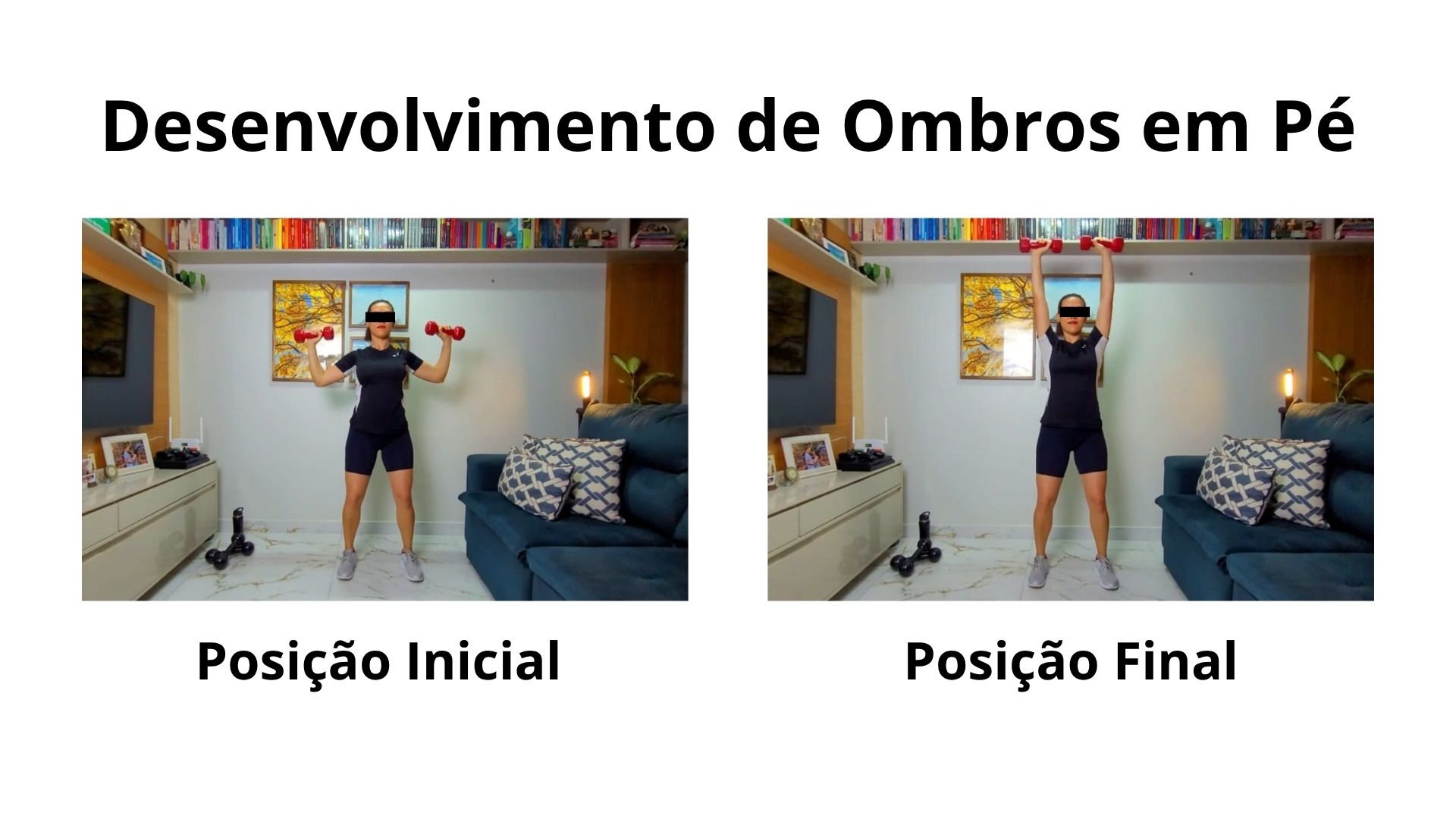


Initial position Final position

1. Circuit 2 - Supine chest fly (Intensity progression: load increase)


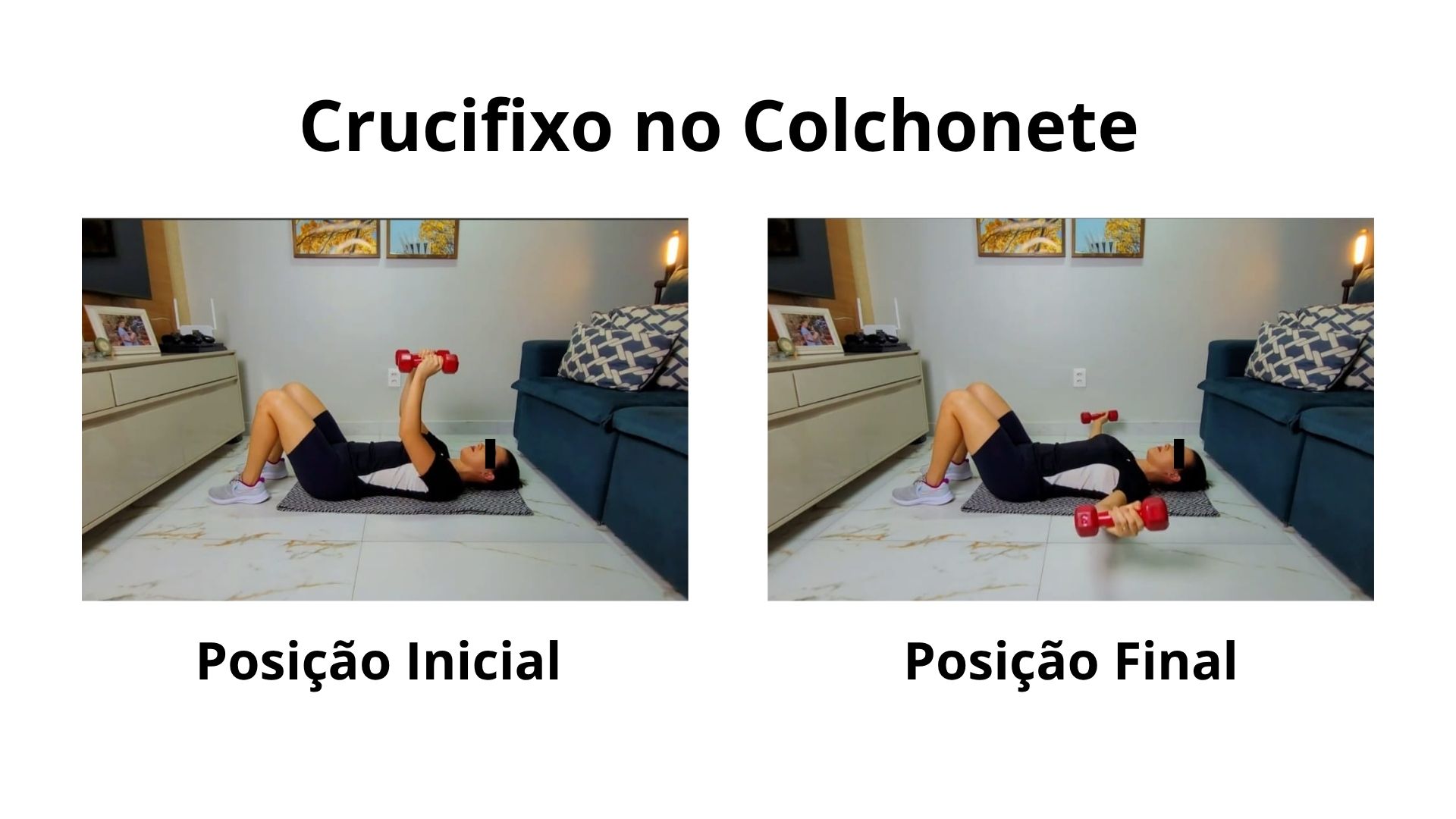


Initial position Final position

1. Circuit 2 - Calf raises (level 1: bilateral)


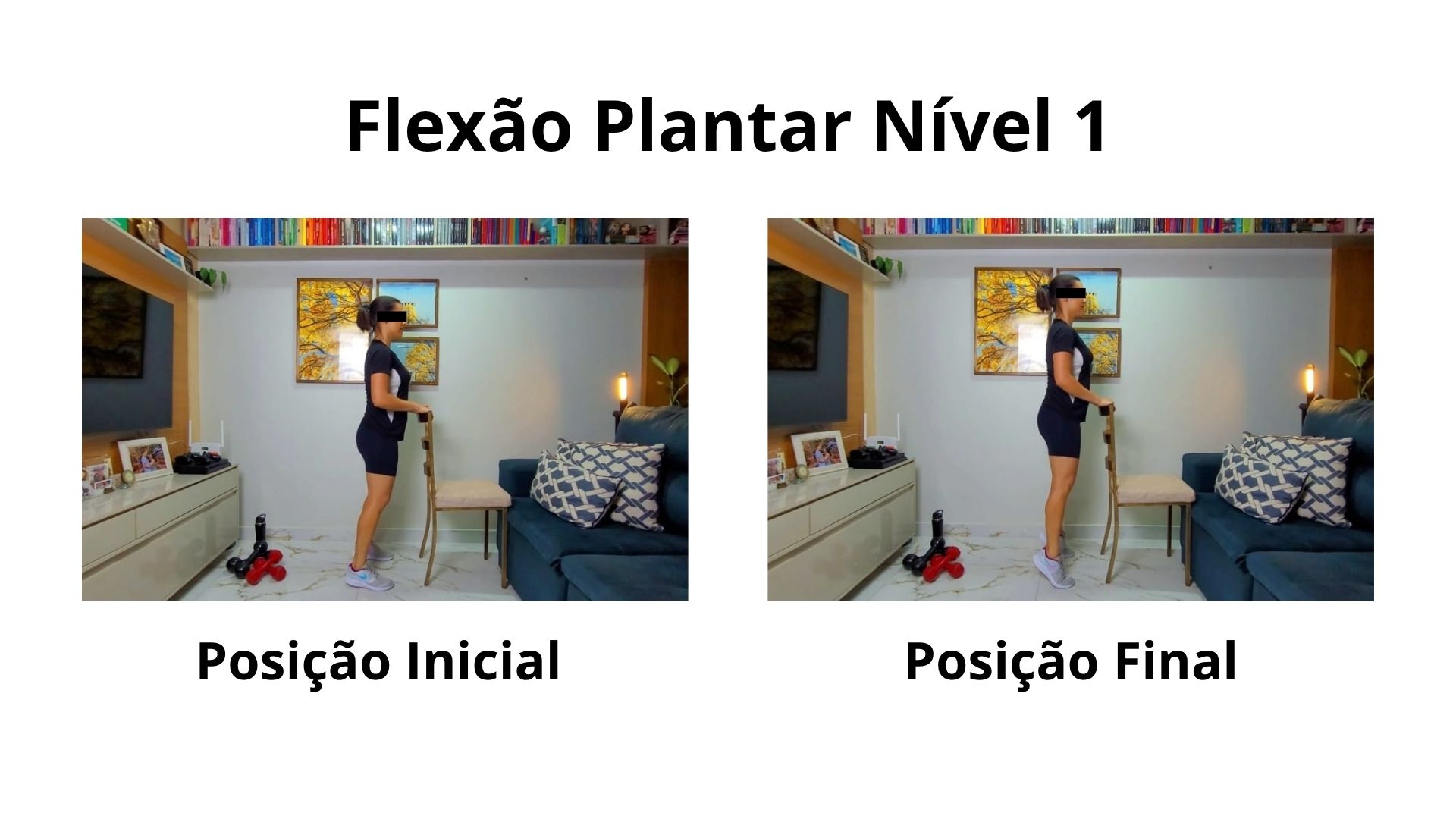


Initial position Final position

1. Circuit 2 - Calf raises (level 2: unilateral)


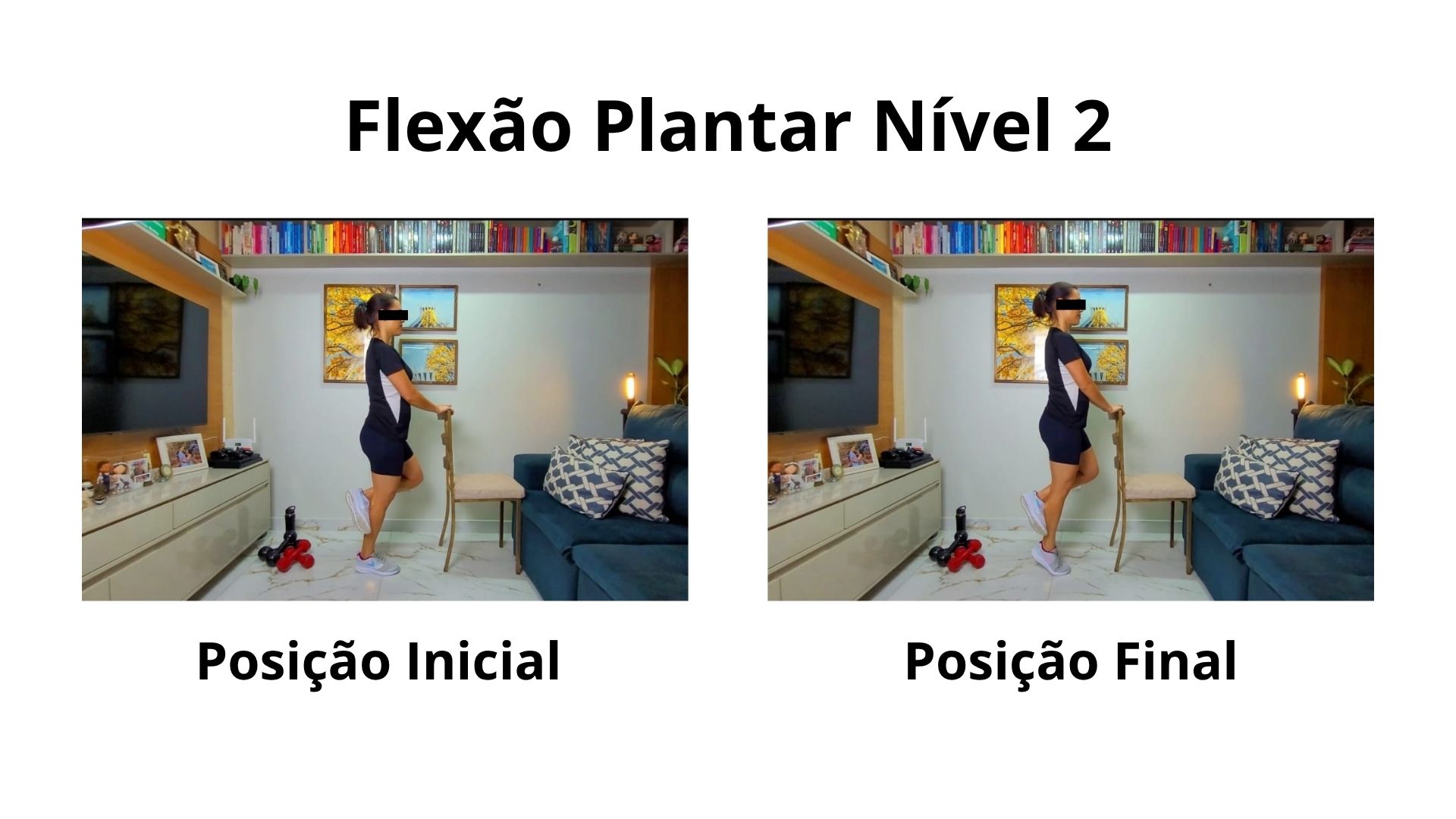


Initial position Final position

1. Circuit 2 - Plank on mat (Intensity progression: by duration)

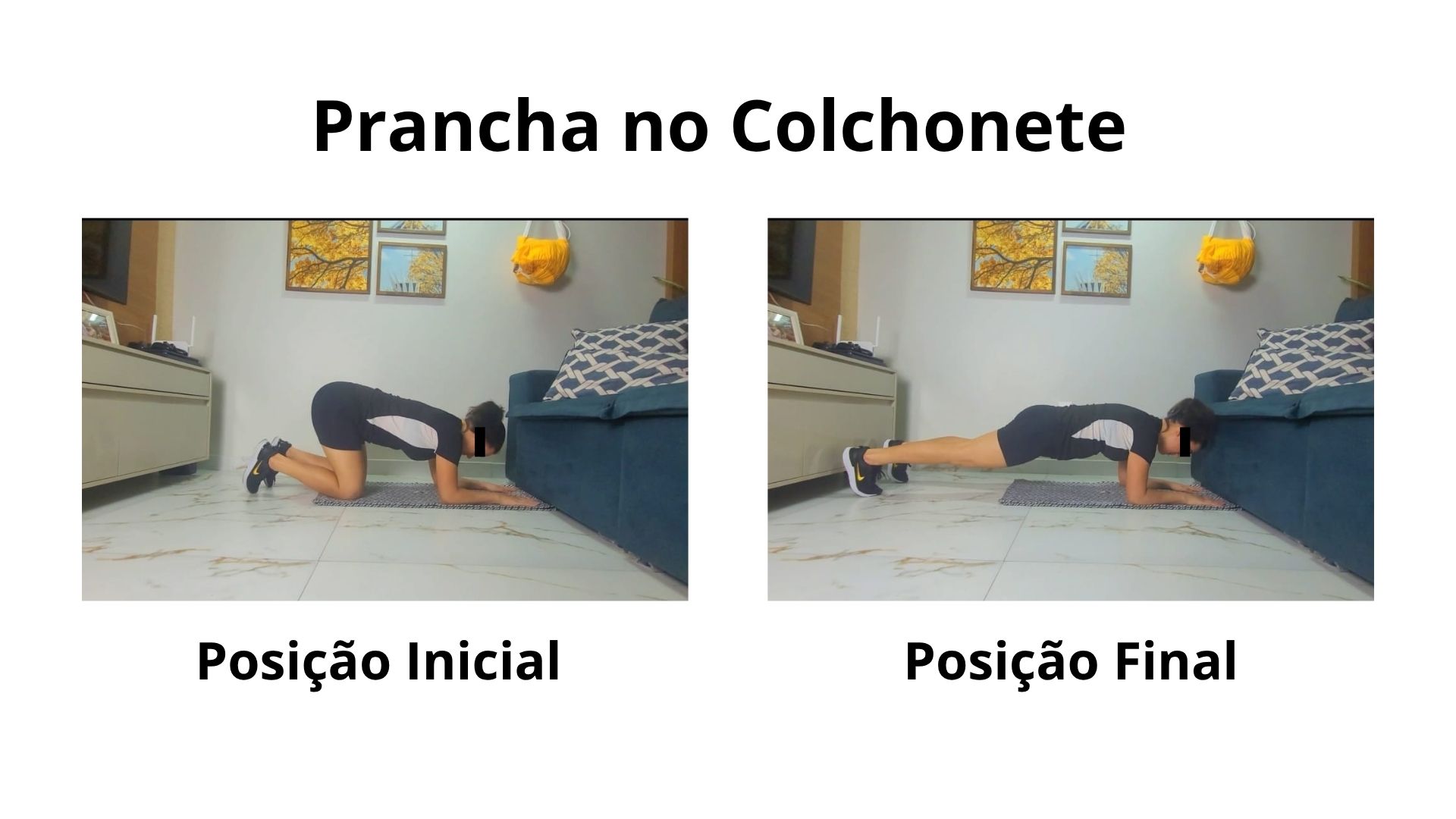


Initial position Final position

1. Circuit 3 - Triceps kickback (Intensity progression: load increase)


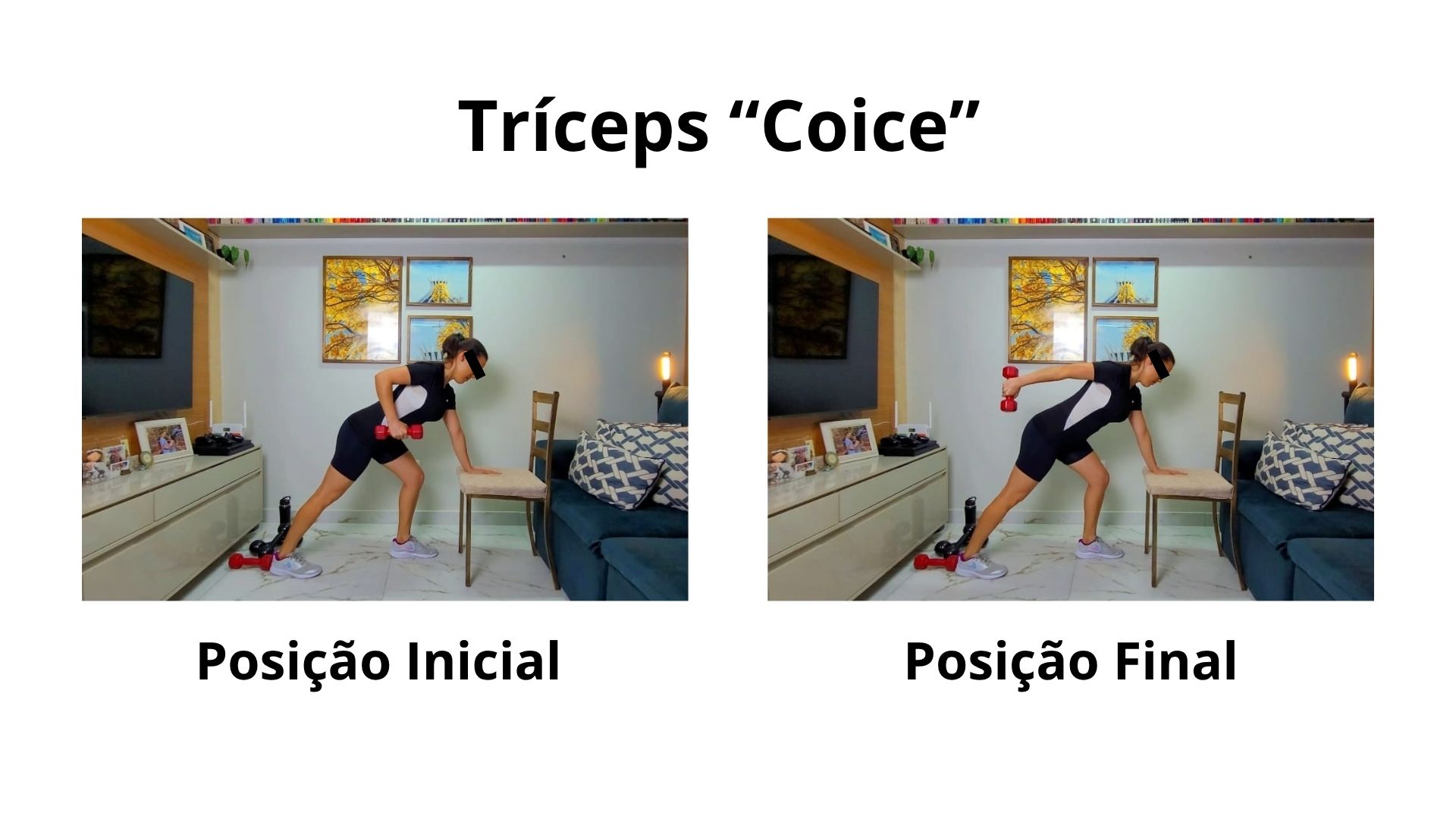


Initial position Final position

1. Circuit 3 - Bicep’s curl (Intensity progression: load increase)


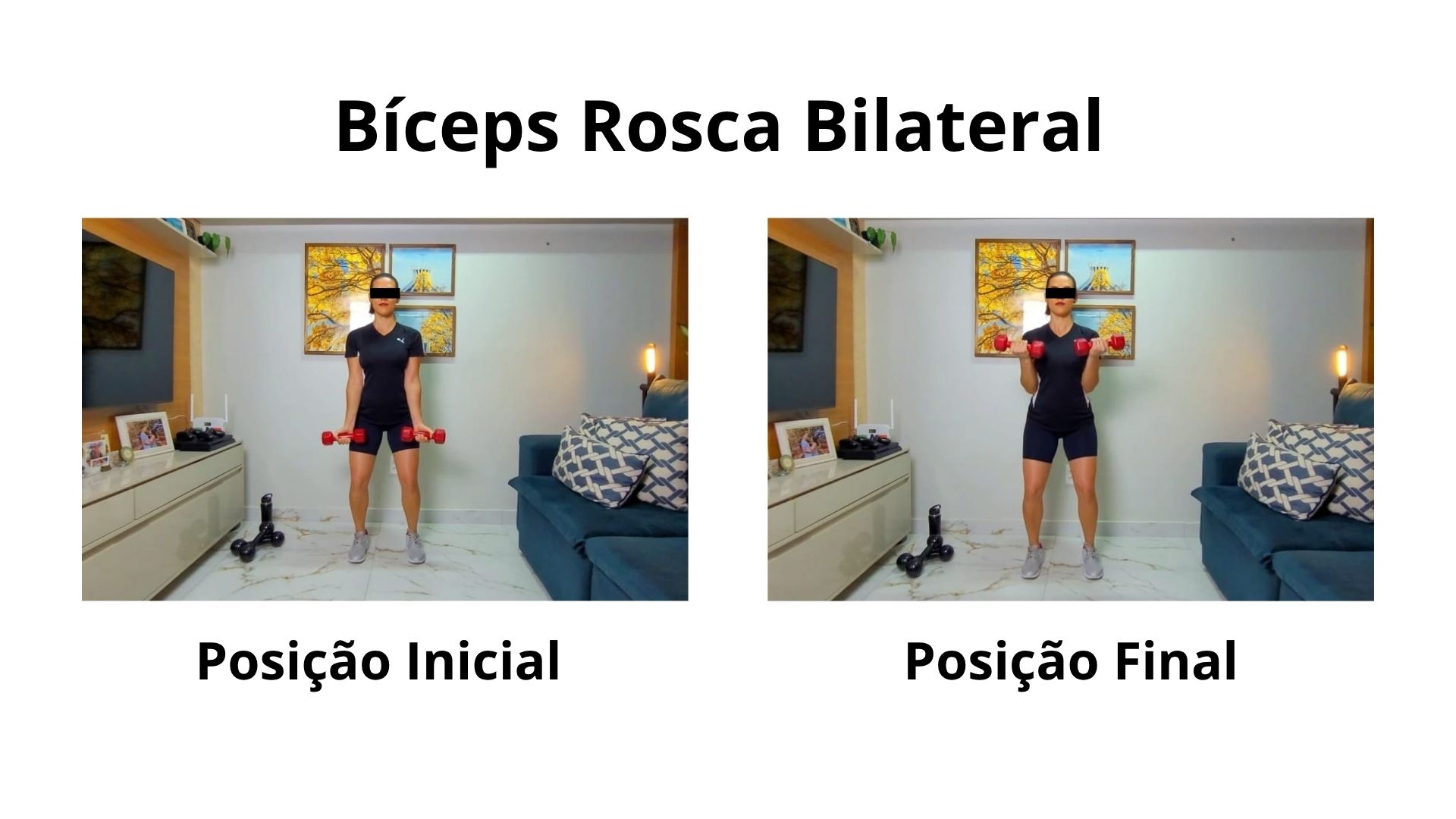


Initial position Final position

1. Circuit 3 - Hip raise on mat (level 1: bilateral)


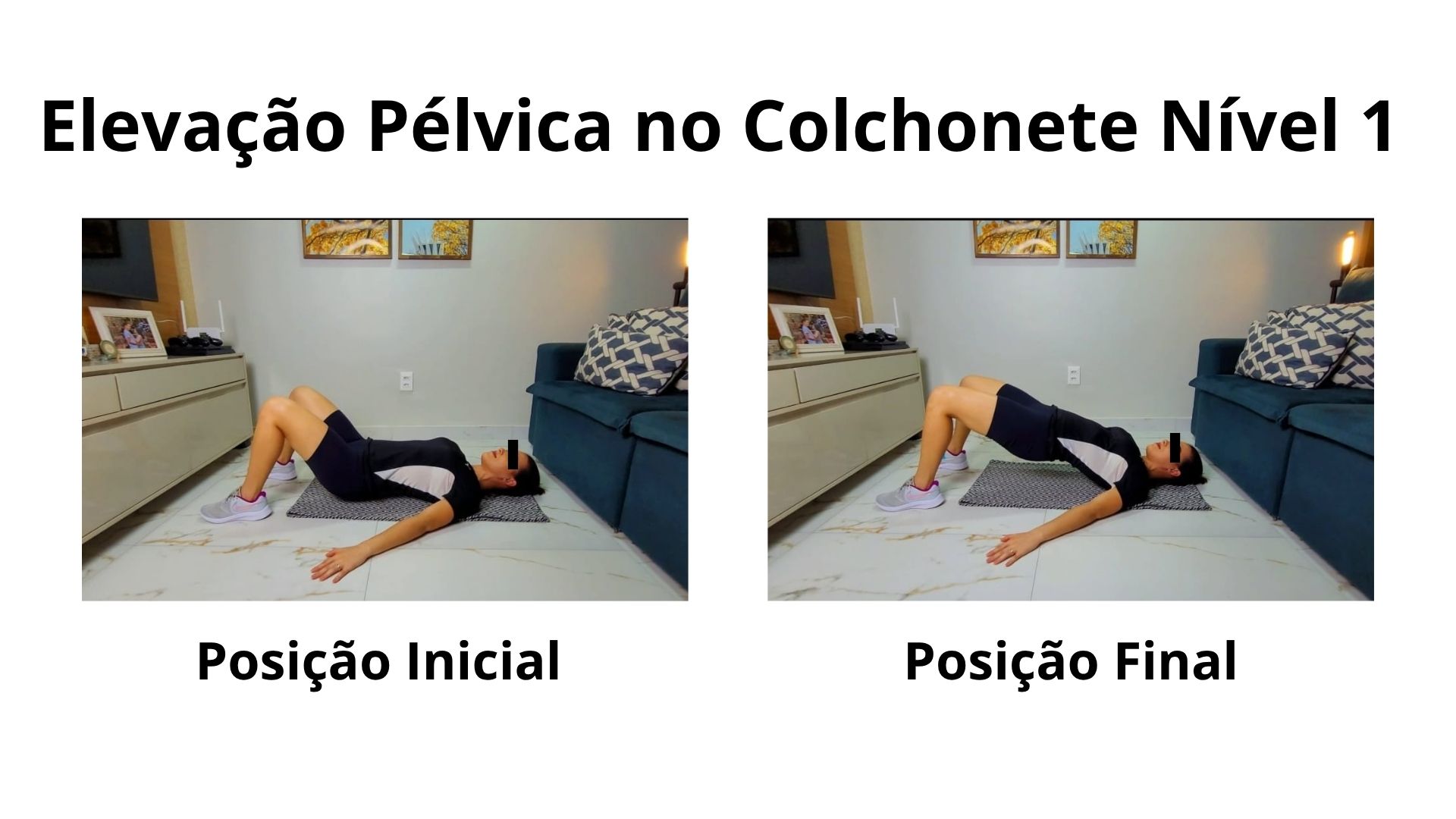


Initial position Final position

1. Circuit 3 - Hip raise on mat (level 2: perpendicular arms)


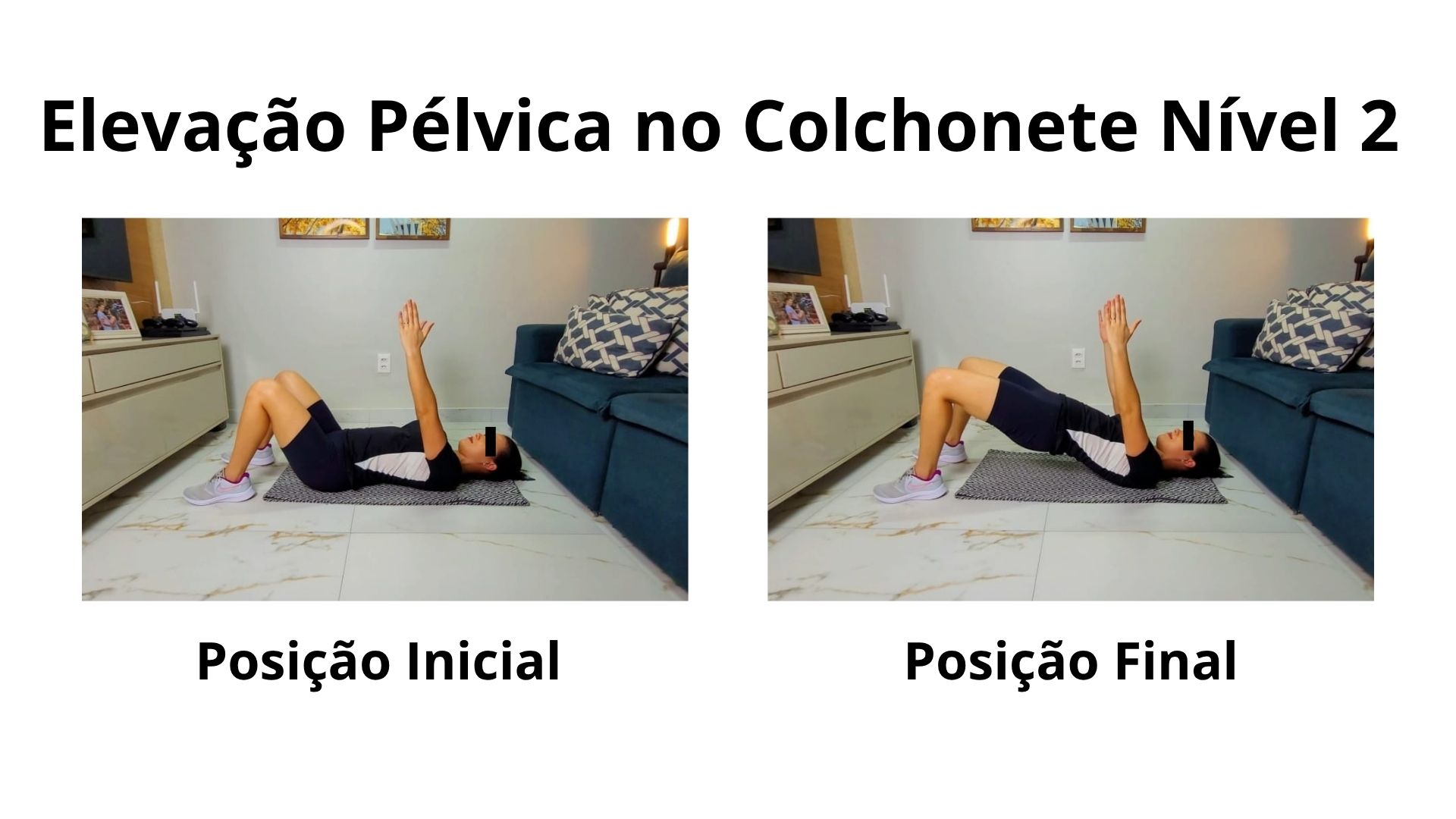


Initial position Final position

1. Circuit 3 - Hip raise on mat (level 3: unilateral)


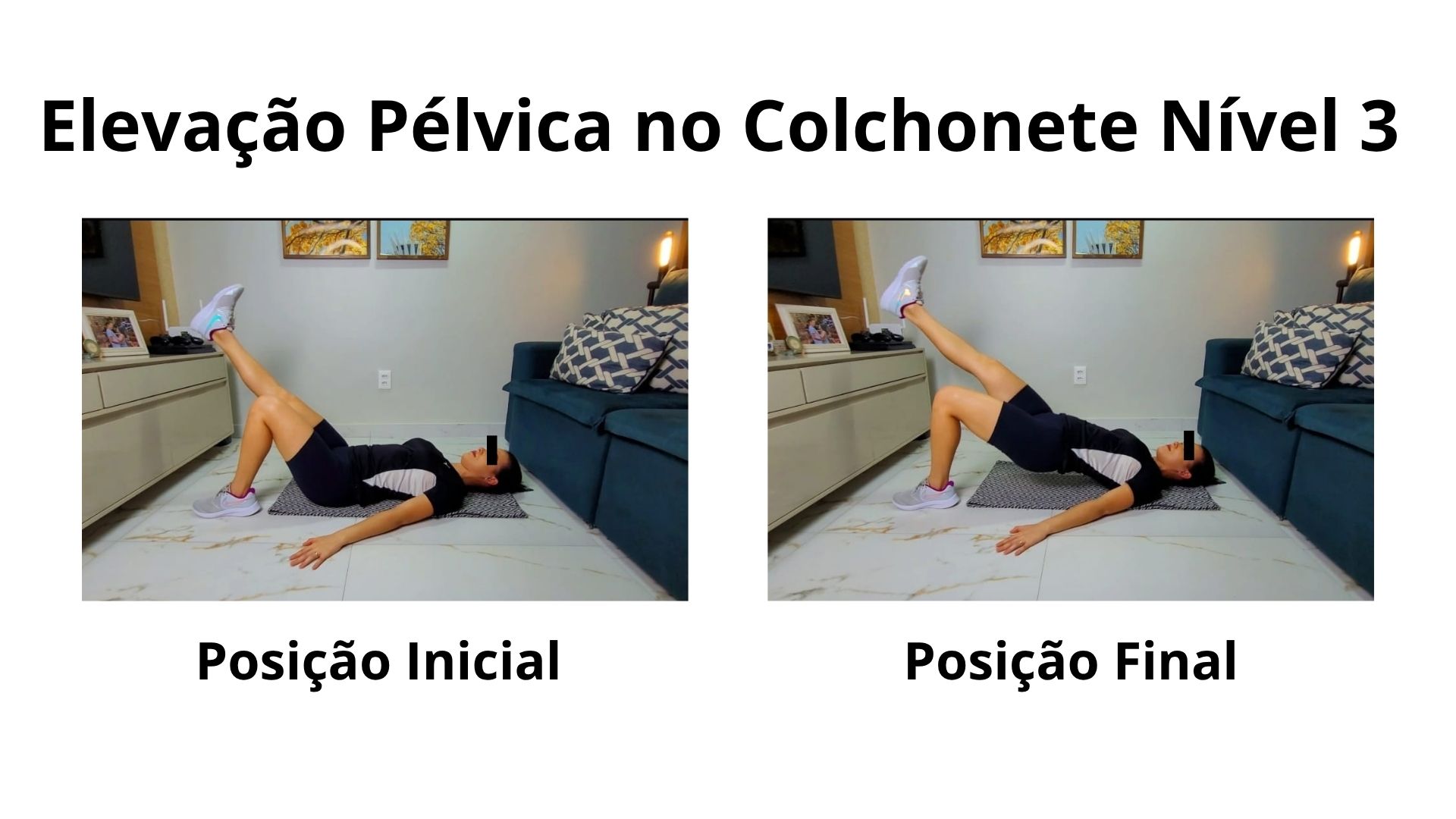


Initial position Final position

Note: the images shown were used with the explicit consent of the individual appearing in the photographs.

## References

1. Ministério da Saúde. Secretaria de Atenção à Saúde. Departamento de Atenção Básica. Guia Alimentar para a População Brasileira [Internet]. 2^a^ edição. Brasília; 2014. Available from: http://189.28.128.100/dab/docs/portaldab/publicacoes/guia_alimentar_populacao_brasileira.pdf

2. Ministério da Saúde Hospital do Coração. Alimentação cardioprotetora: Manual de orientações para profissionais de saúde da Atenção Básica [Internet]. 2018. Available from: http://189.28.128.100/dab/docs/portaldab/publicacoes/alimentacao_cardioprotetora_orien_pro_saude_ab.pdf

3. American College of Sports Medicine. Chapter 10: Obesity and overweight. ACSM Guidelines for Exercise Testing and Preescripcion (10th ed.) [Internet]. 2018. Available from: https://www.academia.edu/36843773/ACSM_Guidelines_for_Exercise_Testing_and_Prescription_10th

4. Robertson RJ, Goss FL, Rutkowski J, Lenz B, Dixon C, Timmer J, et al. Concurrent Validation of the OMNI Perceived Exertion Scale for Resistance Exercise. Med Sci Sport Exerc [Internet]. 2003 [cited 2021 Dec 12];35:333–41. Available from: http://journals.lww.com/00005768-200302000-00024
